# Integration of polygenic risk with single cell methylation data using met-scDRS

**DOI:** 10.1093/bioinformatics/btag751

**Published:** 2026-10-20

**Authors:** Xinzhe Li, Zhuozheng Shi, Kangcheng Hou, Katherine W Eyring, Cuining Liu, Matthew G Heffel, Chongyuan Luo, Daniel H Geschwind, Bogdan Pasaniuc

**Affiliations:** Genomics and Computational Biology Program, University of Pennsylvania, Philadelphia, PA 19104, United States; Genomics and Computational Biology Program, University of Pennsylvania, Philadelphia, PA 19104, United States; Department of Epidemiology, Harvard T.H. Chan School of Public Health, Boston, MA 02115, United States; Department of Neurology, David Geffen School of Medicine, Neurogenetics Program, University of California, Los Angeles, Los Angeles, CA 90095, United States; Bioinformatics Interdepartmental Program, University of California, Los Angeles, CA 90024, United States; Department of Human Genetics, David Geffen School of Medicine, University of California, Los Angeles, Los Angeles, CA 90095, United States; Department of Human Genetics, David Geffen School of Medicine, University of California, Los Angeles, Los Angeles, CA 90095, United States; Department of Human Genetics, David Geffen School of Medicine, University of California, Los Angeles, Los Angeles, CA 90095, United States; Department of Neurology, David Geffen School of Medicine, Neurogenetics Program, University of California, Los Angeles, Los Angeles, CA 90095, United States; Bioinformatics Interdepartmental Program, University of California, Los Angeles, CA 90024, United States; Department of Human Genetics, David Geffen School of Medicine, University of California, Los Angeles, Los Angeles, CA 90095, United States; Center for Autism Research and Treatment, Semel Institute, David Geffen School of Medicine, University of California, Los Angeles, Los Angeles, CA 90095, United States; Department of Psychiatry, Semel Institute, David Geffen School of Medicine, University of California, Los Angeles, Los Angeles, CA 90095, United States; Institute for Precision Health, University of California, Los Angeles, CA 90095, United States; Department of Genetics, Perelman School of Medicine, University of Pennsylvania, Philadelphia, PA 19104, United States

## Abstract

**Motivation:**

Although large scale genome-wide association studies (GWAS) have identified many risk variants for psychiatric traits their functional implication remain largely unknown. One potential mechanism for their risk is through cellular context-dependent epigenetics such as methylation. Previous methodologies have focused on integrating GWAS with single cell transcriptomics, which allowed identification of within-cell-type heterogeneity in disease risk association. We extended this framework to provide a method called met-scDRS that integrates GWAS with single cell epigenomics and investigates how psychiatric disease risk can manifest through methylomics at single cell resolution.

**Results:**

We propose an approach that integrates single cell methylomes with disease associated genes identified from GWAS at single-cell resolution. We found our method to be well calibrated and powered in different scenarios. We then analysed a publicly available single cell methylome dataset of the human brain for 75 different traits and generated disease relevance scores for each cell for each trait. We find high brain specificity with major cell type enrichments congruent with known findings. We identify within-cell-type heterogeneity for psychiatric disease risk along the brain spatial axis as well as subpopulations that are differentially associated with diseases. Finally, we showed that genes with high correlation between methylation levels and disease relevance scores to be highly enriched in specific functional pathways such as synaptic formation and transmission.

**Availability:**

met-scDRS is freely available in GitHub as a python package under MIT license: https://github.com/bogdanlab/met-scDRS. Source code is also available from Zenodo with DOI: 10.5281/zenodo.21841506. All dataset used in this work are publicly available.

## 1 Introduction

Cytosine methylation is a covalent modification of DNA, i.e. found in most CpG dinucleotides in the mammalian genome (Moore *et al*. 2013). In most somatic mammalian tissues, over 70% of CpG sites are methylated while unmethylated CpG sites are enriched in promoters and distal regulatory elements (Jang *et al.* 2017). High levels of non-CpG methylation, or methylation of cytosines outside of CpG dinucleotides, accumulate in human brain tissues during post-natal development (Lister *et al.* 2013). The genome-wide pattern of non-CpG methylation is correlated with gene expression (He and Ecker 2015, Jang *et al.* 2017) and is known to have functional importance in gene silencing (Pinney 2014, Jang *et al.* 2017, Ramasamy *et al.* 2021), gene-environment interaction (Lutz *et al.* 2021), cell type specificity (Ziller *et al.* 2011, Kozlenkov *et al.* 2016), and disease association (Chen *et al.* 2015, Jang *et al.* 2017, Rizzardi *et al.* 2019). There are a few methodologies that integrate GWAS with methylation single-cell data, with most methods focusing on finding heritability in differentially methylated regions (Rizzardi *et al.* 2019) or comparing cell-to-cell methylation differences between cases and controls (Iqbal and Zhou 2023). Such methods often disregard non-CpG methylation, which may have different characteristics than CpG methylation (Ziller *et al.* 2011, He and Ecker 2015, Jang *et al.* 2017). For identifying contexts, such as cell types relevant to disease, current methods focus on utilizing pre-existing marker genes and quantifying their associations with existing GWAS (Calderon *et al.* 2017, Watanabe *et al.* 2019), or directly comparing proportional differences between cases and controls (Lin X *et al.* 2023, Alayoubi *et al*. 2024, Ferrena *et al.* 2024, Phipson *et al.* n.d). In contrast to methylation, transcriptomic signal is routinely overlaid with GWAS signal using methods such as single-cell disease relevance score (Zhang *et al.* 2022) (scDRS) and sc-linker (Jagadeesh *et al.* 2022); since these methods are purpose-built for RNA sequencing, it remains unclear whether they apply to other genomic modalities such as methylation.

Recent technological developments have generated single-cell methylome datasets (Liu H *et al.* 2021, 2023, Luo *et al.* 2022, Tian *et al.* 2023) that resolve cell types, probe disease-associated genomic regions, and methylation-related gene expression changes. Single-cell methylation profiles across multiple CpG sites are routinely aggregated at the level of single-gene methylation patterns (Liu H *et al.* 2021, 2023, Tian *et al.* 2023) to improve signal-to-noise resolution. We expand the single-cell disease relevance score (Zhang *et al.* 2022) framework from RNA-sequencing to methylation data (non-CpG and CpG methylation) to overlay such signal with GWAS. Following standard practice in the single-cell methylation studies (Liu H *et al.* 2021, 2023, Tian *et al.* 2023), where methylation signals are aggregated at the gene levels, our method supports similar aggregation across gene bodies, introns, exons and promoters. Since methylation data have different distributional properties compared to RNA-seq data, we expand the scDRS framework to explicitly incorporate the ratio of methylated to total reads under different normalization schemes while supporting gene length, coverage, and missingness adjustments. The single-cell methylation score assesses hypomethylation near GWAS genes compared to genes matched for variance, methylation levels, and either gene length or coverage density.

We used simulations to find that met-scDRS is well-calibrated when no disease association is present in the data while also being well-powered when a fraction of cells in the experiment are disease implicated. To gauge its performance in a real dataset, we applied met-scDRS with GSE215353 human brain methylation atlas data (Tian *et al.* 2023) across 75 different traits and identified cell-trait associations. Our method showed high specificity toward brain-related traits, demonstrating our method’s true positive rate in real-world settings. On the other hand, we identified many significant cells in brain-related traits that are highly congruent to previous publications (Tian *et al.* 2023) using partitioned heritability. Since our method computes disease association with single-cell resolution, we recover within-cell-type subpopulations with region heterogeneity across traits. For example, in major depressive disorder (MDD), we identified that 18.1%, 9.67%, and 13.3% of L2/3-IT glutamatergic neurons, OPC and MGC cells are significantly associated (FDR corrected *P*-value < .1) respectively, with sub-populations in L4-IT glutamatergic neurons differentially enriched in association. Additionally, our method recovers within-cell-type disease risk variation across dissection regions along the posterior to anterior axis of the brain. We also showed that our method can be extended as a gene prioritization tool in identifying high-confidence drug targets for bipolar disorder (Qi *et al.* 2023) such as PRKCB, CACNA1C, GRIN2A, HTR6, DAGLA, KCNB1 and CACNAB2 as well as finding disease-related functional pathways. To validate our results, we performed stringent robustness analyses across different normalization schemes, and null selection schemes to ensure proper null distribution while also testing our method in a mouse brain dataset, where we identified similar enrichments.

## 2 Methods

### 2.1 Met-scDRS algorithm

From methylation cytosine sequencing, met-scDRS takes gene-level methylation ratios as input where the ratio is defined as methyl-cytosine base coverage divided by the total cytosine base coverage aggregated at designated genomics region. met-scDRS supports four different aggregation regions: (i) promoter, (ii) intron, (iii) exon, (iv) gene body. For promoters, 2000 base pairs (bp) upstream of transcription start site (TSS) and 500 bp downstream of TSS are aggregated for each gene. For introns, distances between exons are counted and the region between TSS and the first exon is excluded from aggregation. For exons and introns, we counted the methylated cytosine and coverage and took the sum across all exons or introns in a gene to aggregate into gene-level fraction. This results in a cell by gene level matrix for each of the aggregation method.

The purpose of aggregation is that it substantially reduces data dimension and sparsity. Then, met-scDRS uses the following steps to obtain the preprocessed ratio$\;X_{c,g}^{\prime }$: (i) met-scDRS calculates the inverse fraction of methylated cytosine as$\;1-X_{c,g}\;$where$\;X_{c,g}\;$is the aggregated ratio at cell c and gene g as methylation levels are expected to be anticorrelated to the gene expression. It should be noted that such expectation, albeit supported by known genetic-regulation mechanism (Patil *et al*. 2014), is not always observed (Luo *et al.* 2022). Second, we filter out genes with very low variance (< 5th percentile) for variance stabilization as well as genes without known gene length. Third, we apply an arcsine transformation for additional variance stabilization. After the arcsine transformation, met-scDRS provides two different methods for selecting control genes. First, if the coverage information is available, control genes can be matched by preprocessed ratio, variance, and coverage density ($\sum_{i}(\mathrm{Coverag}e_{ig}/\mathrm{Gene}\;\mathrm{lengt}h_{g}$). However, when coverage for each cell and gene is not available, such as when using processed released data, control genes can also be matched based on gene length, preprocessed ratio, and variance by percentile to obtain 10 × 10 × 10 bins where the total number of genes are equally spread across bins. Finally, we use the existing algorithm detailed in scDRS (Zhang *et al.* 2022) for obtaining cell-wise *z*-scores (met-scDRS) and *P* values. For completion, we expand on how met-scDRS computes *z*-score and *P* values:

1. The met-scDRS take the putative gene set with associated weight as input denoted by$\;W_{g}\;$with g=disease gene1, 2, 3, …, disease gene G and control genes are sampled from genes with matching criteria mentioned previously and repeated B times. This procedure resulted in *B* sets of control genes, with each set having G genes.
2. met-scDRS computes technical noise$\;\sigma_{\mathrm{tech},g}\;$as the standard deviation where$\;g=\mathrm{gene}\;1,2,\ldots ,N_{\mathrm{gene}}\;$in methylation matrix
3. For each cell c, met-scDRS computes raw disease score ($s_{c}$) and B sets of raw control scores ($s_{c,b}^{\mathrm{ctrl}}$). Under the same notation as scDRS with π(g) denoting the disease gene that corresponds to control gene g.
  $$\begin{array}{c} s_{c}=\frac{\sum_{g\in G}\;w_{g}\sigma_{\mathrm{tech},g}^{-1}X_{c,g}^{\prime }}{\sum_{g\in G}\;w_{g}\sigma_{\mathrm{tech},g}^{-1}}\\ s_{c,b}^{\mathrm{ctrl}}=\frac{\sum_{g\in G_{b}^{\mathrm{ctrl}}}\;w_{\pi (g)}\sigma_{\mathrm{tech},g}^{-1}X_{c,g}^{\prime }}{\sum_{g\in G_{b}^{ctrl}}\;w_{\pi (g)}\sigma_{\mathrm{tech},g}^{-1}} \end{array}$$
4. met-scDRS perform alignments to correct for mismatches within bins with the same mean and variance alignment as scDRS (Zhang *et al*. 2022) where
  $$\begin{array}{c} s_{c}\leftarrow s_{c}-\frac{1}{n_{\mathrm{cell}}}\sum_{c^{\prime }=1}^{n_{\mathrm{cell}}}s_{c^{\prime }}\\ s_{c,b}^{\mathrm{ctrl}}\leftarrow {(s}_{c,b}^{\mathrm{ctrl}}-\frac{1}{n_{\mathrm{cell}}}\sum_{c^{\prime }=1}^{n_{\mathrm{cell}}}s_{c^{\prime },b}^{\mathrm{ctrl}})\\ \times \frac{\sum_{g\in G_{b}^{\mathrm{ctrl}}}w_{\pi (g)}\sigma_{\mathrm{tech},g}^{-1}}{\sum_{g\in G}w_{g}\sigma_{\mathrm{tech},g}^{-1}}\sqrt{\frac{\sum_{g\in G}w_{g}^{2}\sigma_{\mathrm{tech},g}^{-2}\sigma_{g}^{2}}{\sum_{g\in G_{b}^{\mathrm{ctrl}}}w_{\pi (g)}^{2}\sigma_{\mathrm{tech},g}^{-2}\sigma_{g}^{2}}}\\ s_{c}\leftarrow \frac{s_{c}-{\hat{\mu }}_{c}^{ctrl}}{{\hat{\sigma }}_{c}^{ctrl}};s_{c,b}^{ctrl}\leftarrow \frac{s_{cb}^{\mathrm{ctrl}}-{\hat{\mu }}_{c}^{\mathrm{ctrl}}}{{\hat{\sigma }}_{c}^{ctrl}};b=\{1,\ldots ,\;B\;\mathrm{control}\;\mathrm{sets}\} \end{array}$$
5. And cell-wise *P* values as
  $$p_{c}=\frac{1+\sum_{c^{\prime }=1}^{n_{cell}}\sum_{b=1}^{B}\;I(s_{c}\leq s_{c^{\prime },b}^{\mathrm{ctrl}})}{1+n_{\mathrm{cell}}B}$$
6. Convert cell-wise *P* values into *z* score (met-scDRS) under normal distribution.

To summarize, the procedure partitions genes based on matched properties for computing a null distribution. For further details, we refer to the original scDRS publication (Zhang *et al.* 2022), as well as our publicly available source code (https://github.com/bogdanlab/met-scDRS).

Although the principle of scoring is similar to scDRS, met-scDRS differs in the following ways: (i) met-scDRS is designed with the distribution of fractions in mind. It is tailored specifically to methylation ratios that are aggregated at different genomic regions. This is possible because we have normalizations specifically for ratios as well as inversion techniques that follow biological intuition. The same is not true for scDRS where data points are assumed to be either in counts or normalized unbound values. (ii) met-scDRS takes gene length or coverage density into consideration and explicitly match control genes based on matching properties; a core mechanism not shared with scDRS. (iii) Memory optimizations are done with respect to float type data whereas in scDRS, optimizations are done with respect to count type data.

### 2.2 Single cell dataset and gene set acquisition

We utilized two single-cell methylomics datasets, where one of them contains the human methylome, and the other contains the mouse methylome under GEO accession number GSE215353 and GSE132489. In GSE132489, single-nucleus methylation cytosine sequencing (snmC-seq) of 92 adult mouse tissues (mouse methylation brain atlas) was downloaded from Gene Expression Omnibus GSE132489 on 08/17/2023. This dataset contains 1 03 982 distinct methylomes that passed QC across 17 dissection regions with 41 major cell types profiled by snmC-seq2. In GSE215353, we downloaded 373 888 distinct single cell DNA methylomes across three adult brains spanning 44 regions profiled by snmC-seq2 (human methylation brain atlas) containing 40 major cell types on March 22, 2024. Additionally, we analysed an atlas-level transcriptomics dataset (Tasic *et al.* 2018) released by AIBS and downloaded in December 2024.

For the gene set scores, we first downloaded the same gene set files derived from 74 UK Biobank traits as in the previously published work (Zhang *et al.* 2022) for met-scDRS. Then, we computed the MAGMA gene set using the same procedure used in the published work (Zhang *et al.* 2022) for autism disorder GWAS (Grove *et al.* 2019) and combined the resulting gene set with the original 74 traits. To describe the processing scheme of autism disorder GWAS in detail, we first downloaded the gene location file and SNP location file from MAGMA (De Leeuw *et al.* 2015) website https://ctg.cncr.nl/software/magma for GRCh37 genome build. We then filtered the SNPs in the autism disorder GWAS to the set of HapMap3 (Altshuler *et al*. 2010) SNPs. Next, using the gene and SNP location files that we downloaded, we used MAGMA to generate a gene annotation file with 10 kb as our window size both upstream and downstream of each gene location. Finally, using the filtered autism disorder GWAS, we computed the MAGMA genes using the *P* values of the SNPs within the GWAS and the total number of individuals participating in the GWAS study. The top 1000 genes from the resulting MAGMA gene set file with their respective *Z*-scores are extracted and aggregated into the 74 UK Biobank trait gene set. Additionally, we added an extra MDD GWAS (Adams *et al.* 2025) using the same procedure to assess the reproducibility of met-scDRS with the same trait but a different GWAS.

### 2.3 Calibration simulations

From the GSE132489 mouse methylation brain atlas dataset, we first filtered to the set of cells that passed QC detailed in publication (Liu H *et al.* 2021) and randomly selected 10 000 cells. We preprocessed these 10 000 cells using the techniques detailed in the met-scDRS algorithm section to obtain$\;X_{c,g}^{\prime }$. From the preprocessed matrix, we further randomly selected three sets of genes: (i) genes randomly selected from all the features in the simulation matrix. (ii) Top 25% of genes with the highest fractions. (iii) Top 25% of genes with the highest fraction variances. To account for the effect of gene sizes during simulation, sets of 100 genes, 500 genes, and 1000 genes were selected. Next, we permuted the GWAS weights by randomly selecting one trait out of the downloaded 74-trait gene set file and then randomly selecting the top 100, 500, and 1000 Z-scores respectively to pair with the simulated gene set. We also considered a more stringent threshold for simulating the high mean and variance of genes where genes are selected from (i) the top 5% of genes with the highest fraction, (ii) the top 5% of genes with the highest fraction variance using the same procedure mentioned above. We repeated all simulations with 100 replicates to estimate the standard deviation between simulations. In total, we have 100 replicates × 3 gene set sizes × 6 simulations for calibration simulations.

### 2.4 Causal simulations

Using$\;X_{c,g}^{\prime }\;$obtained in the previous null simulation, we perturbed the methylation values in two different ways: (i) fixed overlap and (ii) fixed effect size. For fixed overlap, we first selected 500 cells and 1000 genes from$\;X_{c,g}^{\prime }\;$randomly as causal genes and causal cells. Out of these 1000 genes, only 500 genes (fixed 50% overlap) were chosen from human ortholog genes present in the gene set file of the height trait. The remaining genes were then chosen randomly from all genes present in$\;X_{c,g}^{\prime }$. For these 500 cells and 1000 genes, their values in$\;X_{c,g}^{\prime }\;$were multiplied by 0.6 to 0.8 at 0.1 increments, as well as from 0.8 to 0.9 at 0.01 increments to observe met-scDRS behavior across a range of perturbations. The perturbed$\;X_{c,g}^{\prime }\;$is capped at 1 such that all values after perturbation remain bounded between 0 and 1 to retain the processed data in the fraction space. For the fixed effect size causal simulation, we varied the overlap previously held at 50% while keeping the perturbed effect size constant at 0.7 times the original$\;X_{c,g}^{\prime }$. We explored a range of overlaps from 10% to 60% at 10% increments. For the causal simulation, the gene weight from the height trait was used. We repeated all simulation settings 50 times and quantified power as the percentage of perturbed cells correctly identified as significant (FDR corrected *P*-value < .1) by met-scDRS.

### 2.5 Donor and coverage effect simulations

To assess the robustness of our method to donor and coverage variation, we introduced donor-specific noise into the count and coverage matrices used to derive methylation fractions. For each individual i, we drew a donor-specific noise scale τ,


$$\tau_{i}∼|N(0,m)|$$

where m denotes the overall magnitude of the donor and coverage effect. For every cell and gene belonging to individual i, we added noise, drawn separately for the count and coverage matrices, to their original values:


$$\varepsilon ∼N(0,\tau_{i})$$

Noise was added only to entries with non-zero coverage. We then recomputed the methylation fraction as count divided by coverage and bounded the resulting fractions to [0,1]. This procedure yields donor-specific noisy methylation fractions, where the induced perturbation scales inversely with coverage. We repeated the process 50 times for each noise magnitude m∈{1,2,5,10,15,20,40}.

For the downstream calibration and causal simulations, we fixed the perturbation effect to 0.7 and the overlap to 0.5, keeping all other settings as described above, and used the resulting donor-specific noisy methylation fractions as input.

### 2.6 Region effect

To evaluate the effect of region-specific methylation signals, we extended the causal simulation to selectively perturb methylation aggregated at exon, intron, and promoter regions for each gene. For this simulation, we took a subset of approximately 50 000 cells (50K subset) from the basal ganglia, hippocampus, and cortex from the GSE215353 dataset and their cell-type clustering result from previous work (Heffel *et al.* 2026). For each of these cells, the originally generated raw AllCools files were downloaded which allowed us to extract the methylation coverage information and to aggregate methylation at intron, exon, promoter and gene-body regions. We tested one out of the three regions at a time. For the tested region, we scaled its methylated counts within the causal cell-by-gene set by an effect of 0.6, 0.7, 0.8, or 0.9, while scaling the counts of the two remaining regions by the background effect of 0.7. We then computed the overall methylation fraction from the perturbed counts and pooled coverage and scored the resulting matrix. We fixed the perturbation overlap to 0.5. Each combination of tested region and region-specific effect was repeated 50 times, for a total of 3 × 4 × 50 = 600 runs.

### 2.7 Sensitivity analysis

Motivated by the fact that CpG and non-CpG methylation can exhibit differential epigenomic characteristics with respect to different genomics regions such as intronic regions, we also conducted a sensitivity analysis to gauge how met-scDRS performs with different regional aggregation methods using the same 50K subset cells. Since coverage can be affected by many technical factors and explicit controls can be important, we provide a coverage-aware preprocessing pipeline: genes with low coverage that are three median absolute deviations away and genes with low variance (< 5^th^ percentile in variance) are removed, cells with less than 50 000 coverage in CpG methylation and less than 100 000 coverage in non-CpG methylation are removed. The following covariates are computed for each cell: methylated cytosine log count, log coverage, and missingness (number of genes that have no coverage) for each cell. For CpG methylation, since the methylation count and methylation coverage are strongly correlated (> 0.99), we drop methylation count from regression to avoid collinearity. Both CpG and non-CpG methylation have control genes matched by mean, variance, and coverage density. For comparison, we compared the coverage-aware preprocessing with the coverage-unaware version where covariates only contain only the log fraction for each cell and control genes are only matched by mean, variance, and gene length. Next, we also provide comparison between methylation aggregated at different genomic regions. For the purpose of this comparison, the coverage-aware preprocessing scheme is used across different aggregated regions. We provide a comparison for the coverage-aware preprocessing scheme with two other processing schemes. (i) control genes matched by mean, variance, and gene length (baseline) and (ii) control genes matched by mean, variance, and gene length with the global methylation fraction for each cell regressed out (coverage-unaware correction).

### 2.8 Met-scDRS application on non-CpG methylation on human methylome data

For real-world application on human single-cell methylomes, we used publicly available GSE215353 (Tian *et al.* 2023) data. We downloaded the preprocessed methylation matrix and did not deploy additional quality control and inherited the cell type labels from the original publication’s metadata for downstream analysis. The dataset contains non-CpG methylation ratios as proportion of total reads with methylated cytosine followed by cytosine, thymine, and adenine aggregated at the gene body. We applied the met-scDRS to the dataset using the 75 traits GWAS gene set file with global methylation as covariates and control genes matched by mean, variance, and gene length. We obtained the cell-wise met-scDRS disease *z*-score (met-scDRS) and *P* values for all traits. We corrected the *P*-value using FDR correction within each of the traits and quantified the number of significant cells (FDR corrected *P*-value < .1).

We visualized the proportion of significant cells in each of the cell types as well as the met-scDRS in UMAP space using the original publication provided coordinates. Using the average met-scDRS for each cell type in each region, we mapped the dissection regions released in GSE215353 onto the human adult brain reference frame (version 0.1) released by the Allen Institute for Brain Science (Ding *et al.* 2016) (AIBS), summarized in Table S1. It should be noted that there are some ambiguities in the mapping since some of the dissection regions are not present in the human brain region atlas released by the AIBS atlas and some brain regions in the AIBS atlas are so coarse that they contain multiple dissected regions released in the GSE215353 dataset. For regions in the reference frame with multiple mappings of dissected regions from the GSE215353 dataset, we aggregated the entries by taking the average of the multiple mappings and dropped the regions that do not exist in the reference frame or outside the common cortex axis. The resulting average MDD met-scDRS at each region was used as color scale for the visualization and rendered with brainrender (Claudi *et al.* 2021) using Python v3.10.10. For robustness analysis, we visualized the ratio of putative disease genes to the rest of the genes in each gene length, mean and variance bins to make sure there is no over-sampling. We also visualized the null distributions and tested whether they followed a normal distribution.

### 2.9 Met-scDRS application on CpG methylation

Human methylomics data also contain CpG methylation ratios as proportions of total reads with methylated cytosine followed by a guanine. Due to the sparsity of CG base in the genome (Smith *et al*. 2025) and the important role of intronic regions (Anastasiadi *et al*. 2018), we aggregated CpG methylation in intron regions for each gene. To better account for the sparsity, we used a coverage-aware methylation preprocessing pipeline detailed in the sensitivity analysis section where QC and covariate regression are explicitly tuned for coverage. Control genes were also matched by coverage density along with mean and variance to provide additional control. We employed this approach across 75 traits on the 50K subset of cells to reduce computational time.

### 2.10 Gene prioritization with score-to-methylome correlation

To investigate the functional role of genes co-methylated with GWAS disease risk gene, we computed the Spearman correlation between the coverage-aware preprocessed methylation levels and met-scDRS for all 75 traits in the 50K subset cells from the human methylome. To prevent the prioritized genes themselves from participating in the disease relevance score computation. We opted for a leave-10%-genes-out procedure. We randomly divided the methylation matrix into ten distinct folds, each containing 90% of the genes of the original methylation matrix. For each fold, we computed the disease score using genes retained in the subset. Then, we evaluated correlation between disease relevance score and methylation level for the remaining putative genes in the 10% holdout genes. Hence, each gene’s correlation coefficient is evaluated on disease relevance scores computed with the gene excluded. For each of the 75 traits, we selected the top 100 genes with the highest Spearman correlation coefficients as our foreground gene sets. We interpret each Spearman correlation as each gene’s met-scDRS contribution and provide a means to quantify how disease-relevant each gene is. For the background gene set, we used the set of 1000 GWAS MAGMA genes present in the coverage-aware preprocessed matrix; i.e. we used the disease gene set input for met-scDRS as a way to control for background putative genes that are already known to be associated with disease. Using this approach, we can know what genes are important for disease association on top of a set of MAGMA genes which are known to be broadly associated with the trait *a priori*. Using the selected foreground and background sets, we tested for pathway enrichment within the C5 ontologies released by GSEA (Subramanian *et al.* 2005, Liberzon *et al.* 2011, 2015) with ClusterProfiler (Yu *et al.* 2012). For each of the 75 traits, we visualized the top 10 over-represented gene ontologies and the genes from the foreground that are the most associated with ontologies in a network diagram. Additionally, we also identified any prioritized genes in bipolar disorder that are drug targets.

### 2.11 Met-scDRS application on GSE132489

To assess the reproducibility and robustness of our method, we also tested our method on the mouse methylation brain atlas dataset. We first filtered to the same set of cells using the quality-filtering criteria listed in the publication’s metadata file (Liu H *et al.* 2021), kept the cell type labels as in the original publication, and did not do any additional classification. We applied met-scDRS to the dataset using the GWAS gene set file containing 75 traits for non-CpG methylation. Within each disease and each modality, we then performed FDR correction and quantified the number of significant cells (FDR corrected *P*-value < .1). Using the output from the met-scDRS, we visualized the proportion of significant cells that are in each dissection region, cell classes, major cell types, and cell subtypes. We also visualized met-scDRS *z*-score of significant cells in the UMAP space provided in the original publication.

### 2.12 Comparison between met-scDRS on the methylome with scDRS on transcriptome

Motivated by an interest in understanding how methylome can encode different information compared to the transcriptome. We set out to compare met-scDRS applied to the human methylome with scDRS applied on a human single-cell transcriptomics dataset. We used an atlas-level transcriptomics dataset released by the Allen Institute for Brain Science (AIBS) and computed the original disease risk scoring (scDRS) for Alzheimer’s disease, bipolar disorder, MDD, schizophrenia and height. For met-scDRS, we used the coverage-aware preprocessed 50K subset data whereas, for transcriptomics, a simple preprocessing scheme with donor id and sex regressed out was used. We visualized and compared the proportion of significant cells in each cell class for the MDD and Alzheimer’s disease.

## 3 Results

### 3.1 Single cell disease scoring for methylation is calibrated and powered in simulations

Using the single-cell disease relevance score framework (Zhang *et al.* 2022), the input to the approach is a single-cell methylation dataset and a disease gene set (from GWAS) to compute a disease relevance score and *P*-value for each cell in the data (Fig. 1). Following the recommended practice to reduce sparsity in single-cell methylation data (Iqbal and Zhou 2023), we first tested calibration and power using methylation aggregated at the level of gene bodies (Liu H *et al.* 2021, 2023, Tian *et al.* 2023) (Methods). We investigated the calibration of met-scDRS under different simulation scenarios: random gene selection, highly hypomethylated gene selection, and high-variance gene selection (Methods). These three scenarios were selected to assess met-scDRS robustness for (i) random genes, (ii) hypomethylated genes, and (iii) genes with high methylation variance. met-scDRS is well calibrated in all scenarios and remains unbiased with different gene set (Figs. 2a, S1 and S2). Next, we investigated statistical power in simulation studies. We calculated power under two different scenarios: (i) varying proportions of false-positive disease genes within the input gene set and (ii) varying hypomethylation levels. Scenario 1 is used to investigate when GWAS become better powered to identify more relevant disease genes, how met-scDRS increases in power. Scenario 2 is used to investigate how met-scDRS gains in power when there is indeed biological increase in hypomethylation at those disease genes. We found that met-scDRS power increased with increasing true positive genes in the disease gene set (Fig. 2b). met-scDRS was well powered (> 90%) even when the perturbed effect size is low (0.7 times the methylation ratio) (Fig. 2c). Combined, our simulation studies show that our method is well powered to identify cells that are causally associated with disease while being well-calibrated even when genes are randomly sampled from high-variance and hypomethylated gene sets.

**Figure 1:**
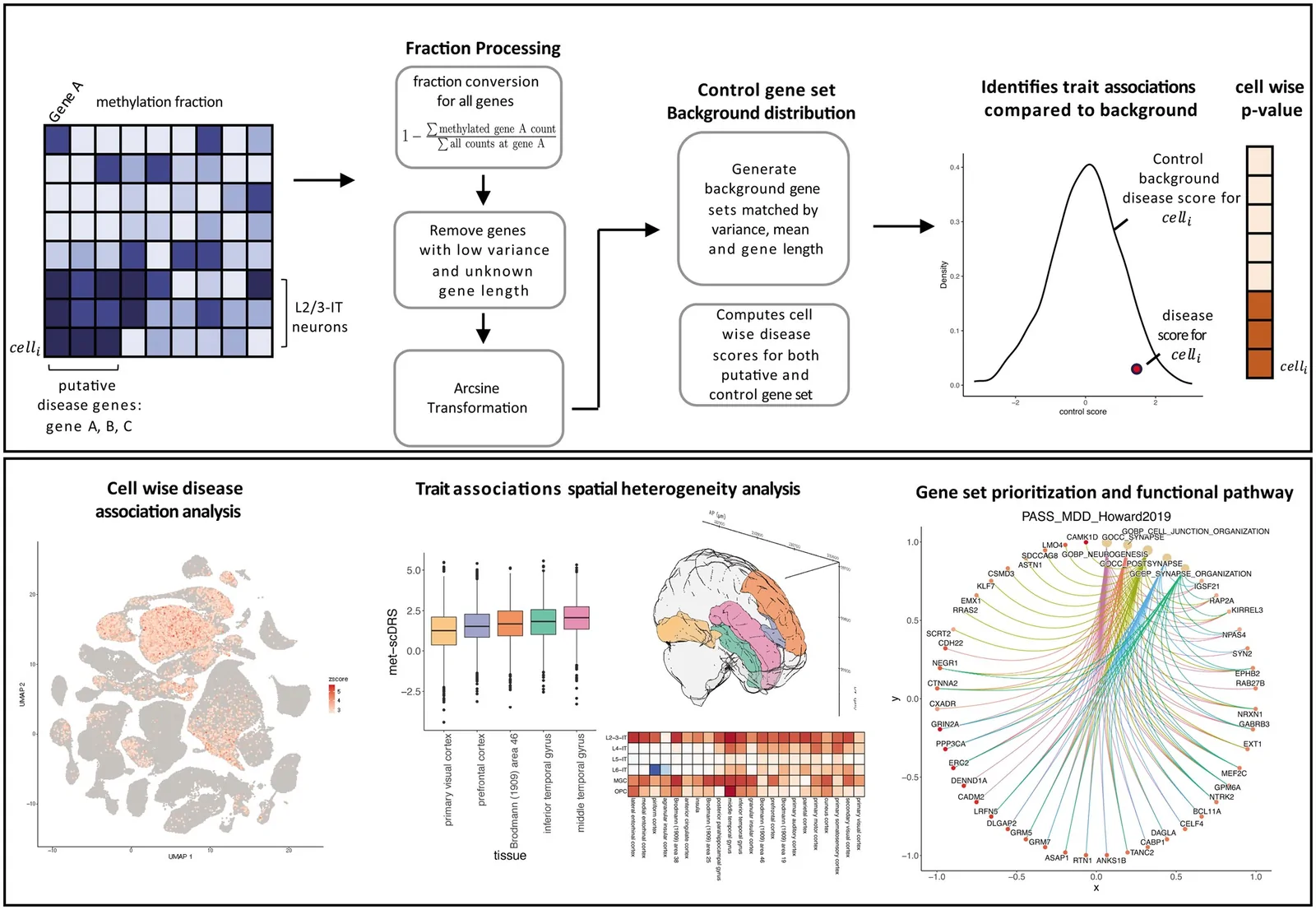
(Top) met-scDRS algorithm, from left to right: gene-level processing schemes, background distribution by sampling, computation of *P* values. Genes with very low variance and no gene length information are filtered for quality control. Using the putative gene set, met-scDRS generates control gene sets with matching properties to compute cell-wise disease relevance score and *P* values. (bottom) Example of a downstream analysis overview from this work. From left to right: UMAP visualization on met-scDRS relevance scores; cell-type-specific spatial heterogeneity analysis across different brain regions; gene ontology pathway analysis by using the prioritized gene sets from met-scDRS.

**Figure 2:**
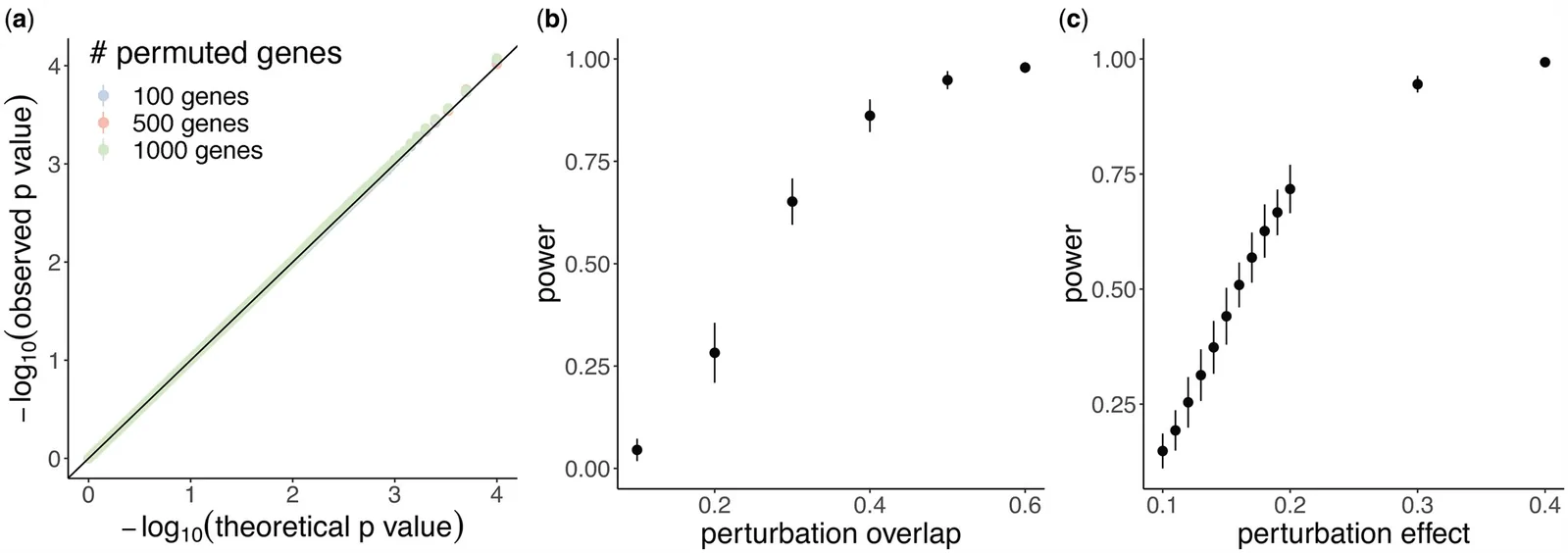
Simulation results for calibration and power. (a) Calibration curve showing observed *P* values against the theoretical *P* values colored by different numbers of putative disease genes randomly selected from a set of genes with > 75th percentile of variance. (b) Power curves showing power computed with overlap from 10% to 60% of the GWAS putative disease genes selected as causal genes, and (c) power curve with perturbation effect computed as (1—effect size) where effect size ranges from 60% to 90% of the original methylation fraction. All confidence intervals are computed as 2 × the standard deviation computed using 50 replicates.

To explicitly test for donor and coverage effects, we also tested calibration and power with respect to donor-specific noise on counts and coverage. At all magnitudes of the donor specific noise tested, met-scDRS performed robustly with no mis-calibration (Fig. S3a). However, power can drop substantially when the variance of the fraction becomes dominated by added noise (Fig. S3 b). We note that this is expected because, as more donor-specific noise is injected, the covariance pattern between methylated fractions becomes dominated by noise, and the true associations are harder to identify. We also conducted simulation studies to probe how power changes if the causal signal comes from a specific genomic region when the met-scDRS has access only to gene-body-level aggregate. This is a likely scenario in real-world settings as gene-body-level aggregate is more accessible and performing aggregation for each cell at each genomic region is computationally expensive and time-consuming. We show that among promoters, introns, and exons, introns are the most sensitive to the potential power changes (Fig. S4). In other words, if the causal association truly comes from introns, met-scDRS can achieve higher power using intron-aggregated methylation. Promoters and exons, on the other hand are less influenced. This is likely due to introns having the largest number of base pairs, and more methylomics alterations are possible, whereas the promoters and exons are much shorter, so the effect is less obvious.

### 3.2 Sensitivity analysis on met-scDRS

Motivated by the fact that CpG and non-CpG can exhibit differential epigenomic characteristics with respect to different genomic regions such as intronic regions, we also conducted a sensitivity analysis to gauge how met-scDRS performs with different regional aggregation methods. We performed met-scDRS with methylomics aggregated using four different methods (promoter, exon, intron, gene body) and computed the pairwise correlation to aggregation at the gene body (Fig. S5a–f). We noticed that in both non-CpG and CpG methylation, aggregating at gene body is the most correlated with aggregation at introns (Spearman correlation = 0.870 and 0.756, respectively). Aggregation at exons for each gene shows moderate Spearman correlation (0.566 and 0.584, respectively) whereas aggregating at promoter regions showed weak correlation (Spearman correlation = 0.233 and 0.275). Next, we used MDD as an example to gauge how different aggregation methods can change the identified significant associations. We observe that met-scDRS identifies a similar number of significant associations (FDR corrected *P*-value < .1) in non-CpG methylation but yields a higher number of significant findings in CpG methylation when aggregated at introns (Fig. S6a and b) in relevant cell types. Together, the results suggest an intron specific effect from CpG methylation that can lead to higher power at identifying significant associations in brain-related traits and methylation aggregation at intron regions is preferred to yield higher power.

Since coverage can be affected by many technical factors and explicit controls can be important, we also gauged the performance of met-scDRS when coverage is explicitly controlled (Methods). We noticed that met-scDRS achieves higher brain specificity and yields more significant findings after FDR correction especially in non-neuronal cells (Fig. S7). This is likely due to the differential distribution between excitatory, inhibitory and non-neuronal cells in coverage (Fig. S8). We also compared met-scDRS across different processing schemes. Globally, we observed a high correlation among different processing schemes (Fig. S9a–d). However, we noticed that in non-CpG methylation, caution should be advised when regressing out global methylation level after matching by mean, variance, and gene length as it may lead to attenuation of signal when sample size is low.

### 3.3 Application of met-scDRS on human brain methylome to understand disease risk heterogeneity

To gauge the performance of met-scDRS in real data, we first deployed met-scDRS to a publicly available human brain methylome dataset across 75 traits for the non-CpG methylome modality. These 75 traits span five different categories: brain traits, immune/blood traits, heart traits, metabolic traits, and other traits (Table S2, Fig. S10). Since the single-cell atlas consists of only brain samples, we hypothesized that met-scDRS would only show a high number of disease-associated cells in the brain-related traits. Indeed, the median number of significant cells (FDR-corrected *P*-value < .1) associated with diseases in each of the five disease categories was enriched for brain-related traits: 3304 (standard deviation = 5263), 1638 (standard deviation = 1959), 726 (standard deviation = 1311), 487 (standard deviation = 337) and 75 (standard deviation = 801) significant cells are associated with brain, metabolic, other, heart, and blood/immune related traits respectively. Next, we quantified, for the three cell classes (excitatory neurons, inhibitory/subcortical neurons, and non-neuronal cells), the average percentage of significant cells in each of the three classes across the five types of traits (Fig. 3a). Brain traits have the highest proportion and variance of significant cells in excitatory neurons and non-neuronal cells, suggesting an abundance of disease associations between hypomethylation and brain-related traits in these cell classes with substantial variation among brain traits. In contrast, blood/immune traits, heart traits, metabolic traits, and other traits all have a minimum proportion of significantly associated excitatory neurons (mean percentage < 1%), showing that met-scDRS identifies excitatory neurons and non-neuronal cells as disease-relevant cell classes associated with brain traits given the dataset. We interpret the result as indicating that met-scDRS detects hypomethylation in relevant cell classes of brain samples as the primary source of signal in brain traits and is well-calibrated to not detect significant associations in non-brain traits while suggesting substantial variation within brain traits and within excitatory neurons and non-neuronal cells (Figs. 3a and S10). Non-neuronal cell types such as MGC, OPC, PC, VLMC, and excitatory neurons L2/3-IT, L4-IT, and L6-IT are broadly associated across many traits to different extents, with substantial variation in proportion across traits (Fig. 3b, Table S2). For example, the L2/3-IT neuronal cell type, although it has broad associations among brain traits, has particularly high enrichment among MDD, years of education and college education traits. Meanwhile, some cell types are more specific in association with diseases such as L6-IT-Car3 and Purkinje neuronal types, which are associated with only education-related traits and Alzheimer’s disease, respectively. We also provide results when the coverage is more explicitly controlled and accounted for (Fig. S7). The overall pattern remains similar to our reported findings where excitatory neurons such as L1-3 neuronal cells provide the most robust signal in psychiatric traits while abundant significant associations can be found in deeper layer excitatory neurons as well. Non-neuronal cells such as OPC, MGC, and PC cells are also abundantly associated with brain-related traits. Overall, we found that at the cell type level, the pattern of enrichment for disease association across traits is similar to what was identified in previous publications by quantifying partitioned heritability over differentially methylated regions (Tian *et al.* 2023).

**Figure 3:**
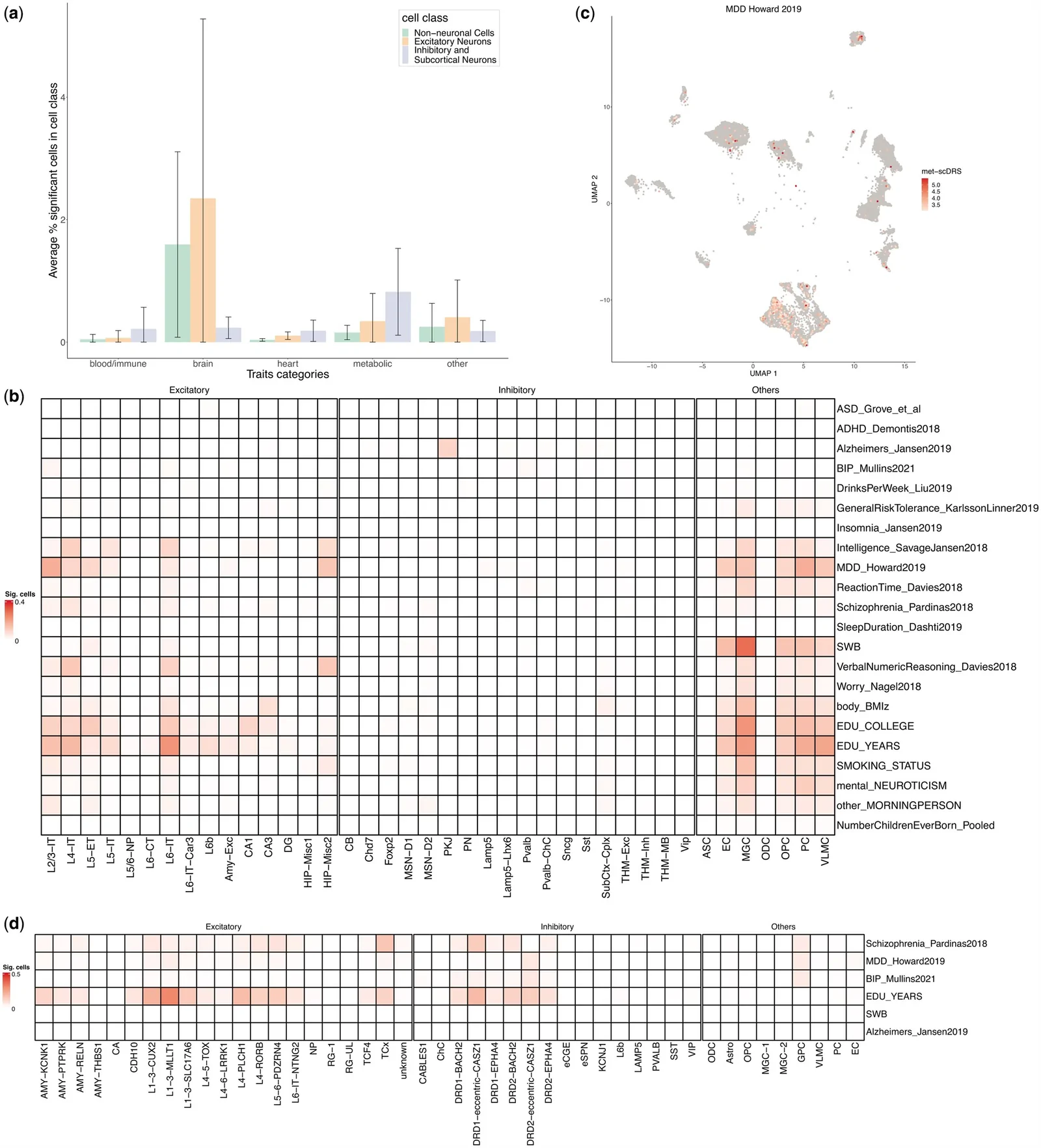
Bar chart (a) showing the average percentage of cells that are significant for each trait categories colored by each cell class. Confidence intervals show ±2× the standard deviation. (b) Heatmap showing the proportion of significant cells in each of the cell types across brain-related traits for non-CpG modality. Cell types are partitioned by excitatory (left), inhibitory neurons (middle), and non-neuronal cell types (right). (c) met-scDRS relevance score on UMAP computed using CpG modality where the significant cells are colored with a gradient from white to red and the non-significant cells are colored in grey. (d) Heatmap showing proportion of significant cells in each cell types for selected brain related traits in CpG methylation.

Since our sensitivity analysis indicated that methylation aggregated at introns can be beneficial, we applied met-scDRS to CpG methylation aggregated at introns through coverage-aware processing for the 50K subset cells. Across all traits, most of the significant (FDR-corrected *P*-value < .1) associations are in brain-related traits (Table S3). Consistent with our observation that L2/3 excitatory neurons are strongly associated with MDD (Fig. 3b–d), we observe 5.66%, 7.41%, and 4.80% of cells in L1/3-CUX2, L1/3-MLLT1 and L1/3-SLC17A6 excitatory neurons were significantly (FDR-corrected *P*-value < .1) associated with MDD, respectively (Table S3). In deep layer neurons, cell types such as L4-RORB and L5/6-PDZRN4 are the most associated, with 8.30% and 15.7% of the cells in cell types are significantly (FDR-corrected *P*-value < .1) associated with schizophrenia. Inhibitory neurons show the greatest number of differences when compared with those in non-CpG methylation. In non-CpG methylation, few significant associations were identified in non-excitatory cell types whereas CpG methylation carries abundance significant associations in the DRD inhibitory neuronal types (Fig. 3b, d). For example, DRD1-eccentric-CASZ1 neurons are the most significantly associated with schizophrenia in inhibitory neurons, with 21.1% of cells in this cell type significantly associated after FDR correction. The rest of the DRD neuronal types such as DRD1-BACH2, DRD1-EPHA4, and DRD2-BACH2 also show abundant associations with >9% of the cells in each of their cell types significantly associated.

We also visualized met-scDRS for each cell in UMAP for MDD, BP, SCZ, and rheumatoid arthritis (Fig. 4a–e) as single-cell resolution allows us to dissect the fine-grained distribution of risk association among diseases. We observed that for different psychiatric traits, the pattern of disease association is different between diseases within the same cell type (Fig. 4a–e). For example, L2/3-IT neurons and L4-IT neurons show clear differences in disease risk among the three psychiatric traits. Driven by this observation, we set out to investigate whether there are subpopulations within the major cell types that are differentially enriched among SCZ, BP and MDD for L4-IT (Fig. 4f–i) and L2/3-IT neurons (Fig. 4j–m). We found that between the three psychiatric traits, subpopulations such as L2/3-IT subcluster 14 have minimal risk associations for BP and SCZ but more substantial association for MDD. With the single-cell resolution, we also observed that within the subcluster 14, there is still a gradient of risk associations for MDD, where cells more clustered to other subtypes (subtype 10 and 16) show higher significance. For L2/3-IT neurons, we also see similar differences among diseases where subtype 1 of the L2/3-IT neurons is more enriched for significant associations in MDD compared to SCZ and BP. We also set out to quantify this pattern across all 75 traits (Figs. S11–S14). Indeed, across a total of 16 L2/3-IT subtypes, we identified subtypes that broadly associate across traits such as subtypes 1, 5, and 10 in MDD, verbal reasoning, and education traits. However, there are also subtypes that show variation between diseases. For example, subtype 8 has particularly high enrichment for disease association in MDD and SCZ (18.0% and 7.5% of cells in this subtype were significant) but is not well associated with any of the educational traits (< 1.5% of cells in this subtype were significant) (Table S4). Additionally, there are subtypes such as subtype 12, i.e. specifically associated with educational traits (9.39% and 10.6% of cells in this subtype were significant for college education and years of education, respectively) that are less observed in psychiatric traits. Similarly, in L6-IT neurons, subtypes 1–7 have a high proportion of significant cells across educational traits but subtypes 4 and 6 have fewer associations in psychiatric traits such as bipolar disorder, SCZ, and MDD. In L4-IT neurons, subtype 4 is enriched in years of education and MDD, but not so in schizophrenia. Our results highlight the utility of having single cell resolution in detecting subpopulations within a cell type that may be particularly enriched in diseases.

**Figure 4:**
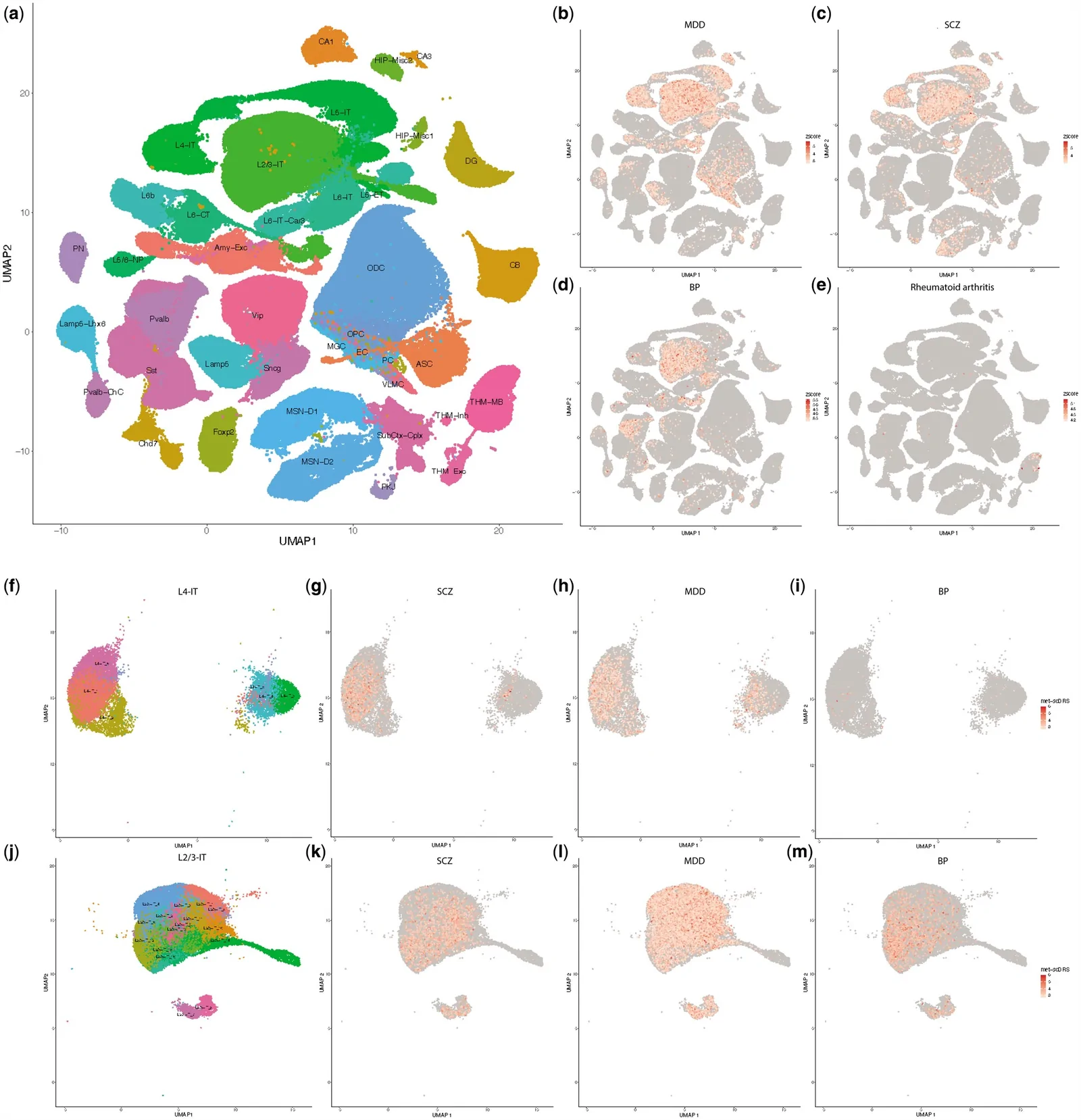
UMAP plot showing non-CpG methylation met-scDRS application on human GSE215353 dataset. (a) UMAP colored by major cell type label (b–e) UMAP colored by met-scDRS for MDD, SCZ, BP, and rheumatoid arthritis disease relevance score respectively. Significant cells are colored in gradient of red color while insignificant cells are colored in grey. (f) UMAP colored by sub-cell type labels for L4-IT neurons. (g–i) UMAP colored by met-scDRS for SCZ, MDD and BP disease relevance score respectively for L4-IT neurons, insignificant cells are colored as grey and significant cells are plotted under the same color scale across diseases. (j) UMAP colored by sub-cell type labels for L2/3-IT neurons. (k–m) UMAP colored by met-scDRS for SCZ, MDD and BP disease relevance score respectively for L2/3-IT neurons, insignificant cells are colored as grey and significant cells are plotted under the same color scale across diseases.

The single-cell resolution of our method also allowed us to probe the relationship between relevance score and spatial origin of the cell. Using MDD as an example, we visualized the average met-scDRS within cell types with respect to different dissection regions across the common cortex axis (Fig. 5a). We noticed that L4, L5, and L6 intratelencephalic neurons all show a higher association from the posterior region to the anterior lateral region of the brain while having much lower associations in the anterior medial regions from the anterior cingulate cortex to the lateral entorhinal cortex. Interestingly, such a trend is not observed in L2/3-IT neurons, MGC, and OPC cells as all three cell types have higher met-scDRS in the anterior medial region of the brain as well (Fig. 5a). We also noticed a gradient of met-scDRS within L2/3-IT neurons from posterior to anterior lateral regions in the brain (primary visual cortex to temporal gyrus) (Fig. 5b and c) while a similar trend is not observed in OPC and MGC cells (Fig. S15). Specifically, L2/3-IT neurons have average met-scDRS scores ranging from 1.22 at primary visual cortex region to 2.02 at middle temporal gyrus while MGC and OPC have average met-scDRS scores ranging from 1.41 and 0.98 at the primary visual cortex to 1.81 to 2.08 at the middle temporal gyrus, respectively (Fig. S15). This result demonstrates met-scDRS is capable at identifying disease-relevant risk heterogeneity across functional annotations of the brain and can be used to highlight specific parts of the brain that maybe more disease associated.

**Figure 5:**
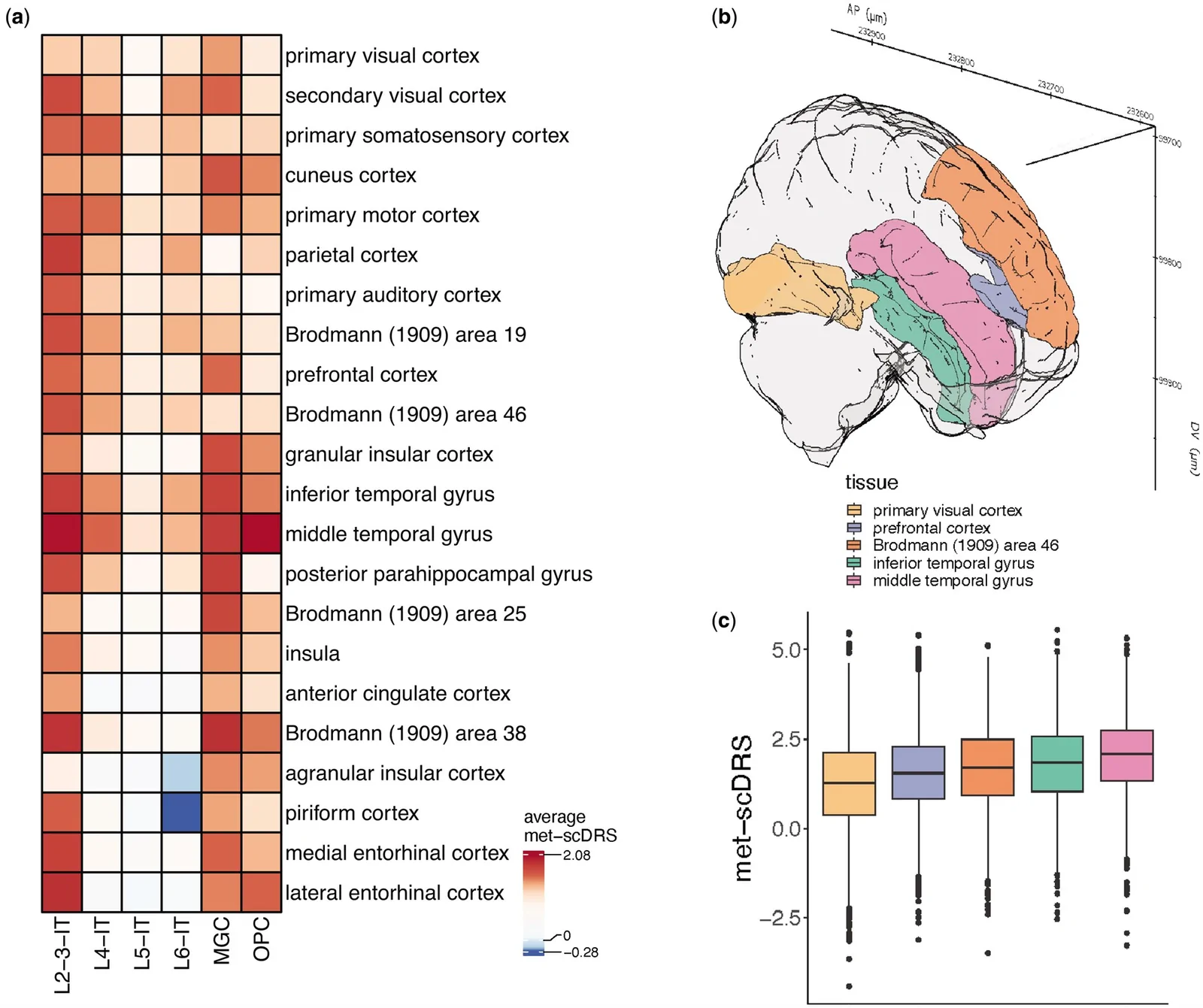
(a) Heatmap colored by met-scDRS in each region for Layers 2–6 intratelencephalic-projecting neurons and MGC, OPC cell types for non-CpG in MDD across the common regional axis. (b) 3D rendering of brain models colored by primary visual cortex (yellow), prefrontal cortex (blue), Brodmann region 46 (orange), middle frontal cortex (purple), inferior temporal gyrus (green). (c) Boxplot of the non-CpG met-scDRS for different regions with the same coloring scheme as brain rendering.

### 3.4 Met-scDRS prioritizes functional genes

We reasoned that genes with co-methylation for GWAS disease-associated genes should be prioritized and analysed to shed light on disease etiology. To this end, we first identified genes with high correlation between the relevance score and non-CpG methylation level and queried their joint biological functions. We illustrate the utility of this approach in two examples concerning bipolar disorder and MDD (Fig. 6a and b). From our gene ontology results, we show that genes prioritized by met-scDRS for MDD and bipolar disorder are both enriched in synapse-related pathways. Specifically, cellular components such as synapse, post-synapses were among the most enriched in both diseases. In addition to the synaptic related functional enrichments, we found that MDD also shows enrichments in neurogenesis-related pathways while bipolar disorder shows enrichment in regulatory-related pathways such as regulation of transmembrane transport and regulation of membrane potential. Combined, our results suggest that regulatory related pathways on neuronal action potential may be more related to the development of bipolar disorder and neurogenesis is perhaps more specific to MDD. Since there is joint enrichment in pathways such synapsis and postsynapse, the dysregulation of these common pathways may explain shared disease etiology between the two disorders. Combined, these results show that met-scDRS provides utility for finding joint pathways between traits as well as distinctions across traits.

**Figure 6:**
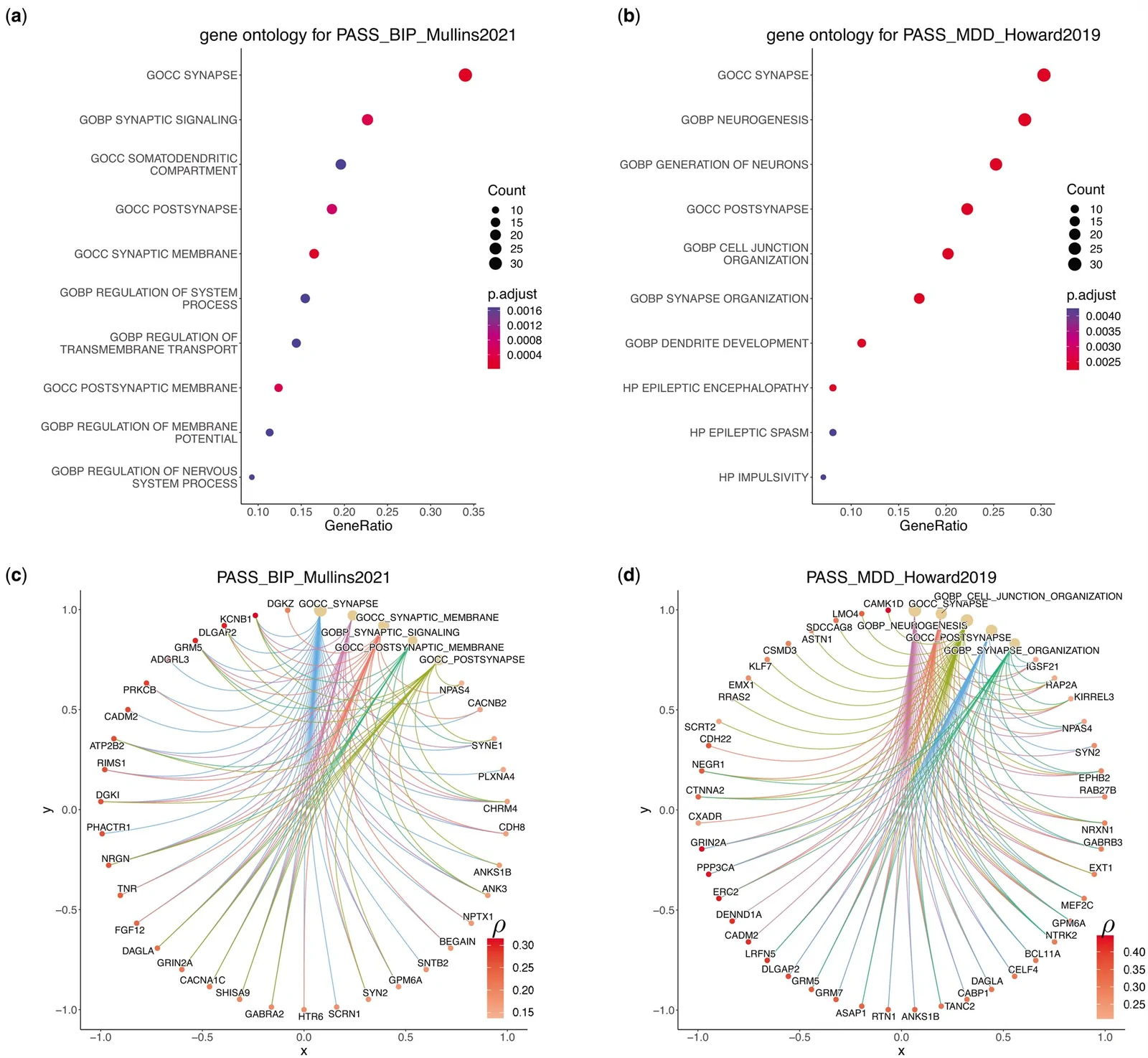
Gene ontology analysis using met-scDRS-prioritized gene set for bipolar (a) and MDD (b). For each of the pathways on the Y axis, the X axis shows the gene ratio between foreground and background; each dot is colored by FDR adjusted *P* value and sized by the number of genes in the foreground that belong to that pathway. Network diagram that shows membership of genes that belong to each of the pathways enriched for bipolar (c) and MDD (d) with lines colored by the pathways and dots colored by spearman correlation between the processed methylation fraction and met-scDRS relevance scores.

Further, we single out genes that are both correlated with disease relevance score and are members of the enriched pathways as they could be prioritized for downstream applications (Fig. 6c and d). For bipolar, we identify genes prioritized by met-scDRS that are viable drug targets. In total, met-scDRS identifies seven bipolar drug targets, of which five out of the seven were identified as tier 1 drug targets and two out of the seven were tier 3 targets for bipolar disorder (Qi *et al.* 2023). For example, PRKCB, DAGLA, GRIN2A, CACNA1C, and HTR6 are genes identified as having high correlation between relevance score and hypomethylation level (Fig. 6c, Table S5) for tier 1 targets, while CACNAB2, KCNB1 belong to the tier 3 targets (Fig. 6c). In addition to finding drug targets, the gene prioritized by our method can also shed light on disease pathology. For example, PCDH9, a gene known for its pathological role in MDD and functional role in synaptic maintenance (Xiao *et al.* 2018, Uemura *et al.* 2022), has high Spearman correlation coefficient (rho = 0.42) (Table S6). Additionally, GRIN2A and CACNA2D1, two protein-coding genes for ion channels that are critical for establishing synaptic transmission (Carvill *et al.* 2013, Beeson *et al.* 2020), are also moderately correlated between their methylation level and met-scDRS with Spearman correlation of 0.44 and 0.45, respectively (Fig. 6d, Table S6). Meanwhile, genes such as CELF2, known for its neuronal differentiation regulatory role(MacPherson *et al.* 2021), also have a high correlation coefficient (rho = 0.53) (Fig. 6d, Table S6) between its methylation and relevance score. Together, met-scDRS provides a rich layer of gene prioritization that can be important for drug targeting and understanding disease biology.

### 3.5 Comparison between met-scDRS on the methylome with scDRS on the transcriptome

We provide a detailed comparison using MDD and Alzheimer’s disease as two example traits to show that biological insights can be gained when both methods are used on different sequencing modalities. First, we observe that MDD shows abundant significant associations (FDR-corrected *P*-value < .1) in excitatory neurons for non-CpG methylation, while few cells reach the significant cutoff after multiple testing correction in scDRS applied to a transcriptomics dataset (Fig. S16a). Meanwhile, in Alzheimer’s disease, 5.36% of the non-neuronal cells are identified as significant (FDR-corrected *P*-value < .1) in transcriptomics (Fig. S16b). When the disease relevant scores are visualized at single-cell resolution, we found that excitatory neurons in layer 3–4 still present an elevated scDRS in MDD, following a similar trend to that in met-scDRS (Fig. S17a, b, e, f). In Alzheimer’s disease, microglia and astrocytes, two important cell types in disease progression (Deng *et al.* 2024) shows strong elevation of relevance scores in scDRS on transcriptomics without clear patterns in met-scDRS on methylomics (Fig. S17c–f). This is because non-CpG methylation is specific to neuronal cell types and non-neuronal cell types are methylated to a lesser extent (Lister *et al.* 2013). This result clearly implies that it is advantageous to identify disease associations not in a single sequencing modality but in both as different sequencing modalities can reveal different sources of biological insights.

### 3.6 Met-scDRS is robust in real world applications and in null specification

To gauge the reproducibility of the methodology toward the same trait but from a different GWAS, we tested a secondary set of GWAS for MDD. We report that the two MDD GWAS have similar met-scDRS (Spearman correlation = 0.7). Additionally, the same set of cell types such as L2/3-IT, L5-IT, L6-IT, OPC, and MGC cell types are strongly enriched in both MDD GWAS (Fig. S18a  and  b). In addition to the gene set reproducibility, we also tested how our findings in met-scDRS can be replicated in the mouse brain atlas. We first investigated its association with MDD and found a similar pattern of neuronal cell types enriched for disease association (Fig. S19a and b). Similar to what we observed in the human atlas, superficial layer L2/3-IT neurons and deeper layer L5-PT and L5-IT neurons showed some of the most significant associations, representing 18.9%, 16.2%, and 6.76% of the total number of significant associations. Also similar to the human atlas results, non-neuronal cells such as OPC and MGC show abundant enrichments in significant associations with MDD, representing 5.18% and 7.55% of the total number of significant associations. To understand brain specificity, we also replicated the same 75 traits in mouse data (Fig. S20, Table S7) and identified similar cell types showing enrichments in significant associations with brain traits. For example, 6.05%, 7.08%, and 18.1% of the OPCs are significant in bipolar disorder, SCZ, and reaction time, respectively while 8.56%, 4.45%, and 14.5% of the MGCs are significant in bipolar disorder, SCZ, and reaction time, respectively. Additionally, deep layer L5 pyramidal tract (PT-L5) cells show the strongest enrichment in significant associations across brain traits, where 6.90%, 10.5%, and 3.90% of the PT-L5 neurons are significant in bipolar disorder, SCZ, and MDD (Figs. S20–S22). Although L2/3-IT neurons are less implicated, significant associations are still detected in brain traits such as MDD, bipolar disorder, and SCZ. Since the PT-L5 neurons are a specialized form of L5-ET neurons (Baker *et al.* 2018), we consider that the pattern of significant association in human at the cell-type level is well recapitulated in mice.

To ensure robustness of the specified null, we first investigated the effect of control-genes matching schemes on our results. To this end, we compared met-scDRS between using mean, variance, and gene length for control gene selection and using mean and variance only for both human and mice across 75 traits (Figs. S23–S25). We report that when gene length is properly controlled for, the cell type enrichment becomes more brain-specific (Figs. S23 and S24). Specifically, the blood traits and other traits became much less enriched for significance within brain cells after the genes are explicitly matched. Some brain-related traits also decreased in enrichment, likely representing a decreased pleiotropic effect after explicitly matching for gene length, making the null distributions less biased toward brain genes. Analysis using mouse data achieved similar results (Figs. S25 and S26). Overall, this suggests that the putative disease genes are well controlled by mean, variance, and gene length with benefits for brain specificity. We also investigated the effect of normalization schemes on met-scDRS. Using a subset of human data, we found that the normalization techniques generally do not have a strong effect at the level of major cell type associations across 75 traits (Figs. S27–S30). However, they result in more robust null distributions, which is important because met-scDRS requires Monte Carlo sampled nulls to follow the same location-scale family. Using a subset of mouse data, we quantified the number of nulls that are not normally distributed significantly after Bonferroni correction under different normalization schemes (arcsine normalization, library size normalization, logit normalization and without normalization). We identified that when not normalized or under library size normalization, a substantial number of null distributions significantly (Bonferroni-corrected *P*-value < .05) deviate from a normal distribution (rounded mean across traits ± s.d: 3526 ± 275 cells, 4092 ± 334 cells, respectively). Under arcsine and logit normalization, 6 ± 7 cells and 3 ± 3 cells, respectively, violate the assumptions significantly. When applied to the full human atlas dataset for MDD, we found only 18 out of 3 73 888 nulls significantly deviate from a normal distribution when normalized by arcsine transformation. Since the arcsine normalization has a variance-stabilization property (Lin and Xu 2020), we recommend it as a default for met-scDRS.

Next, we set out to investigate the robustness of the null distribution. Using MDD as an example, we investigated the background nulls formed by the control gene sets to gauge if the null distributions are well-sampled. This is important because met-scDRS uses control genes to formulate a null distribution to compute cell-wise *P*-value and a high percentage of disease genes in any gene bin leads to deflated power. To this end, we quantified the disease-gene-to-total-genes-in-bin ratio (fg/bg ratio) (Fig. S31). We identified more disease genes that are longer in length, an expected result because brain genes are generally longer, but no oversampled control gene (maximum fg/bg ratio = 23%) in particular mean variance, and gene length bins for the MDD gene set. For completeness, we also visualized the kurtosis and skewness of sampled nulls grouped by cell type (Figs. S32 and S33) and concluded that pooling nulls after alignment on the first two moments is sufficient.

## 4 Discussion

We present met-scDRS as a statistical tool that finds polygenic disease associations at single-cell resolution and prioritizes disease-implicated cells and genes. Using simulation studies, we show that our method is well-powered and calibrated under a variety of different scenarios. Using real data analyses, we found that met-scDRS provides biologically insightful associations in both human and mouse datasets, prioritizes drug targets and functional pathways, and identifies associations complementary to transcriptomics.

In applying our method to a real world dataset, we identified similar cell types enriched for psychiatric traits compared to existing publications (Tian *et al.* 2023). Since met-scDRS processes data at single-cell resolution, it additionally identifies subpopulations and gradients of risk associations among cells. For example, within the temporal gyrus, a known region with MDD associations (McLellan *et al.* 2018, Liu Y *et al.* 2023), met-scDRS identifies the association gradient in L2/3-IT neurons from middle to inferior temporal gyrus. met-scDRS can also be utilized as a gene prioritization tool to identify drug targets and find genes jointly conferring disease-relevant functional pathways, allowing further research into disease etiology within and across diseases. Since non-brain traits usually do not involve brain tissue while brain-related traits do (Ongen *et al.* 2017), we consider the lack of signal in non-brain-related traits to suggest that met-scDRS is well calibrated in real-world scenarios. An added benefit to our method is its utility for both CpG and non-CpG methylation modalities. Since CpG and non-CpG differ in their target methylation cell types (Patil *et al*. 2014), having the capacity to infer disease association in both profiles allows researchers to (i) utilize the under-researched non-CpG methylation modality (Ramasamy *et al.* 2022, Titcombe *et al.* 2022) and (ii) gauge functional convergence and divergence in the respective sequencing profiles.

Given that CpG and non-CpG methylation capture distinctive regulatory effects that are cell-type and genomic-region specific, and substantial variation in coverage is identified across sequencing modalities in different cell classes, it is important to have aggregation strategies tailored to different sequencing modalities with respect to cell type heterogeneity. One advantage of met-scDRS is that it supports multiple genomic aggregations (promoters, introns, exons, and gene bodies). Using sensitivity analysis conducted in real data, CpG methylation showed robust associations with brain-related traits when using methylation aggregated at introns, whereas non-CpG methylation showed similar performance when aggregated in either introns or gene bodies. Since non-CpG methylation is overall less abundant in non-neuronal cells (Lister *et al.* 2013), we recommend using non-CpG methylation mainly for neuronal-cell-type analysis in gene-body aggregation as a robust default. On the other hand, CpG methylation has higher methylation abundance in non-neuronal cells and clearly benefits from aggregation in introns during our sensitivity analysis. As a result, we recommend using CpG methylation for identifying non-neuronal-cell-type associations to brain traits when aggregated across introns. The flexibility in met-scDRS supports differentiated feature selection when making biological discoveries.

We conclude with both the limitations and future work for our approach. First, there is the possibility of strong pleiotropic effects for traits, such as atrial fibrillation, for which met-scDRS can show abundant significant associations within a dataset sequenced from brain cells. Although atrial fibrillation is classified as a heart-related trait, it has a strong neuronal basis in which the mechanism of the disease could be attributed to the excitability of the atrial cardiomyocytes (Pellman and Sheikh 2015, Andersen *et al*. 2021). Such a pleiotropic effect could manifest in met-scDRS as significant associations since the genes associated with atrial fibrillation with respect to such excitability (Andersen *et al*. 2021) could also be particularly perturbed in methylation in brain regions. This demonstrates that false positives could arise with pleiotropic effects. This limitation is also related to the existence of false-positive genes in the putative disease gene set aggregated over GWAS using tools such as MAGMA (De Leeuw *et al.* 2015). Although we have shown that the power of met-scDRS increases linearly with the percentage of true positive genes in the putative gene set, false-positive genes could still lead to a decrease in power. In theory, met-scDRS could also circumvent this limitation by taking in a user-specified gene set such as the SFARI (Abrahams *et al.* 2013) gene set for autism spectrum disorder where putative disease genes are meticulously curated and ranked by publication-related evidence. Such an approach can provide diseases mechanistic insights and allow researchers to understand if their curated gene set using techniques such as forward genetic screening could have an association with diseases via DNA methylation modification. In terms of future directions, one possibility lies in optimizing the methylation aggregation such as using Hi-C data to compute gene scores from MAGMA while matching the aggregation of methylation to gene using the same set of Hi-C results. This approach can perhaps provide additional regulatory regions such as distal repressors and enhancers to identify more biological associations.

## Supplementary Material

btag751_Supplementary_Data

## Data Availability

This study did not generate any new data and contains only analysis done on publicly available datasets. Mouse brain single cell methylome (Liu H *et al.* 2021) dataset is deposited at Gene Expression Omnibus (GEO) under accession number GSE132489 at https://www.ncbi.nlm.nih.gov/geo/query/acc.cgi?acc=GSE132489. Human single cell methylome (Tian *et al.* 2023) dataset is deposited under GSE215353 and can be also downloaded at https://cellxgene.cziscience.com/collections/fdebfda9-bb9a-4b4b-97e5-651097ea07b0. C5 gene ontology pathway file can be downloaded at https://www.gsea-msigdb.org/gsea/msigdb/index.jsp from molecular signature database. AIBS transcription dataset (Tasic *et al.* 2018) can be downloaded from https://portal.brain-map.org/atlases-and-data/rnaseq/human-multiple-cortical-areas-smart-seq. 75 traits gene set files used in this work is deposited at public GitHub repository: https://github.com/bogdanlab/met-scDRS/tree/main/met-scDRS-method/src/met_scdrs/data. Additionally, summary statistics for MDD GWAS (Adams *et al.* 2025) and other psychiatric traits can be both downloaded at https://pgc.unc.edu/for-researchers/download-results/. Source code is also available from Zenodo with DOI: 10.5281/zenodo.21841506. All dataset used in this work are publicly available.
