# Supplementary material for: Integration of polygenic risk with single cell methylation data using met-scDRS
Source: Bioinformatics. 2026 Oct 20;42(10):btag751. doi: 10.1093/bioinformatics/btag751 (PMC13693840; doi:10.1093/bioinformatics/btag751)
Supplement: Supplementary file 1 [file btag751_supplementary_data.zip › Supplementary-figure.pdf]

Supplementary figures

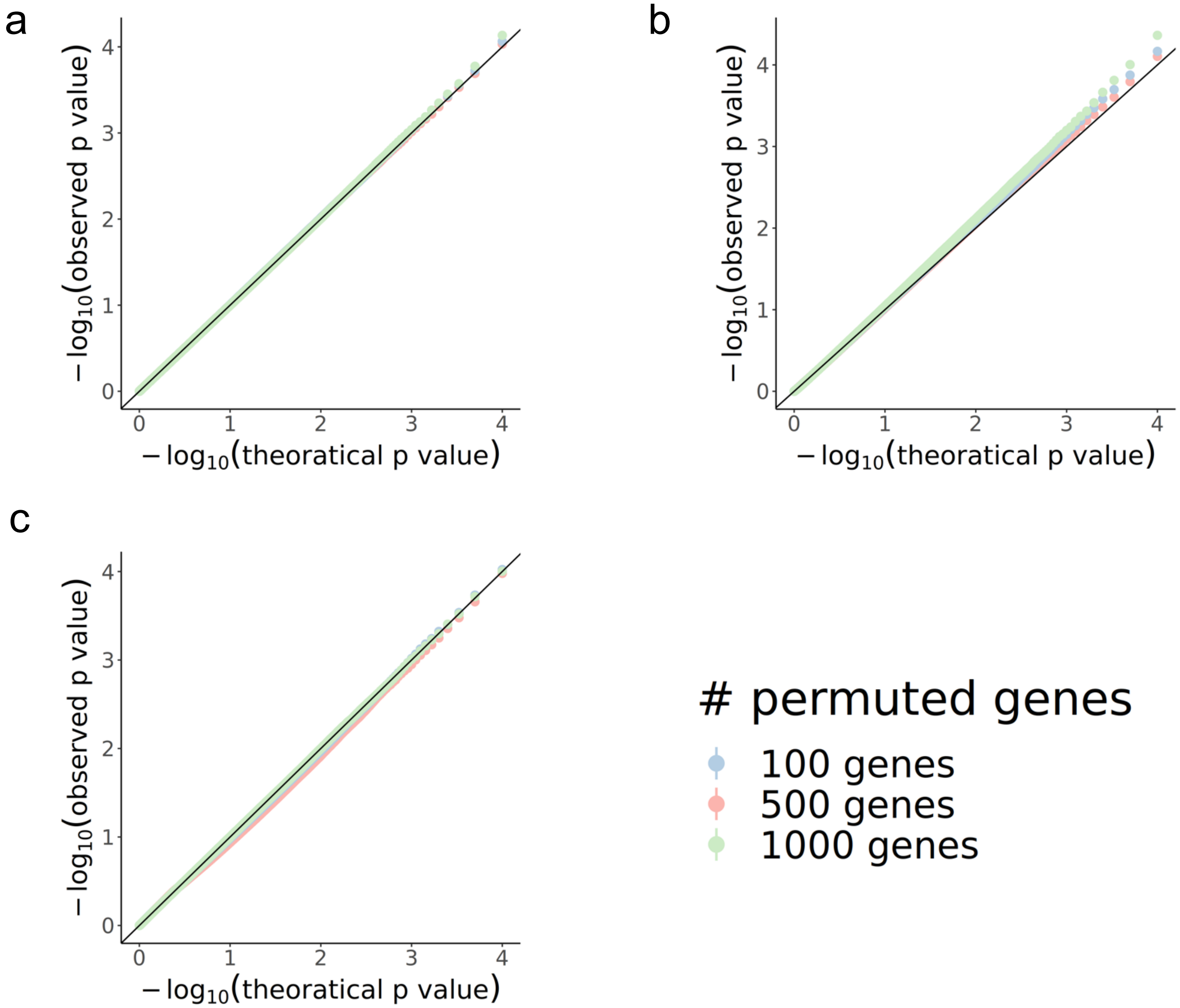

Supplementary figure 1: Calibration curve corresponding to genes draw (a) randomly, genes drew from genes with (b) top 95th percentile in variance and (c) 95th percentile in inverse fraction($X'_{c,g}$). All calibration curves are colored by number of permuted genes within the simulated gene sets. Met-scDRS is run with gene weight option set to inverse standard deviation

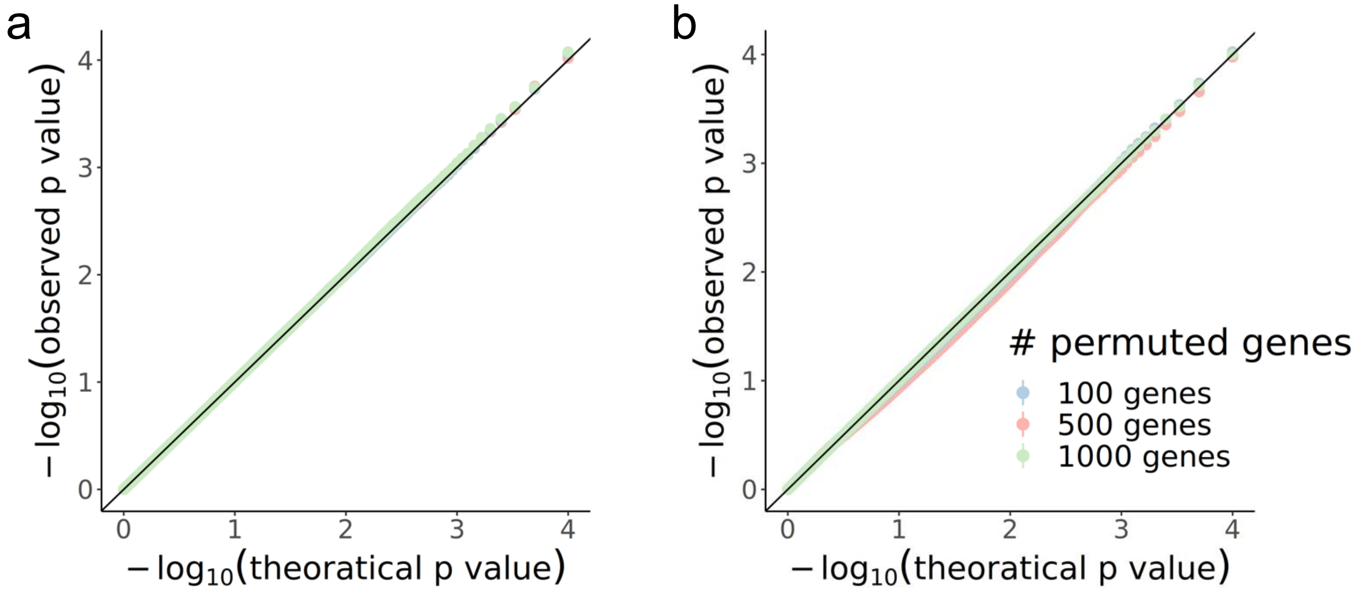

Supplementary figure 2: Calibration curve corresponding to genes draw from (a) top 75th percentile in variance and (b) 75th percentile in preprocessed fraction$(X'_{c,g})$. All calibration curves are colored by number of permuted genes within the simulated gene sets. Met-scDRS is run with gene weight option set to inverse standard deviation

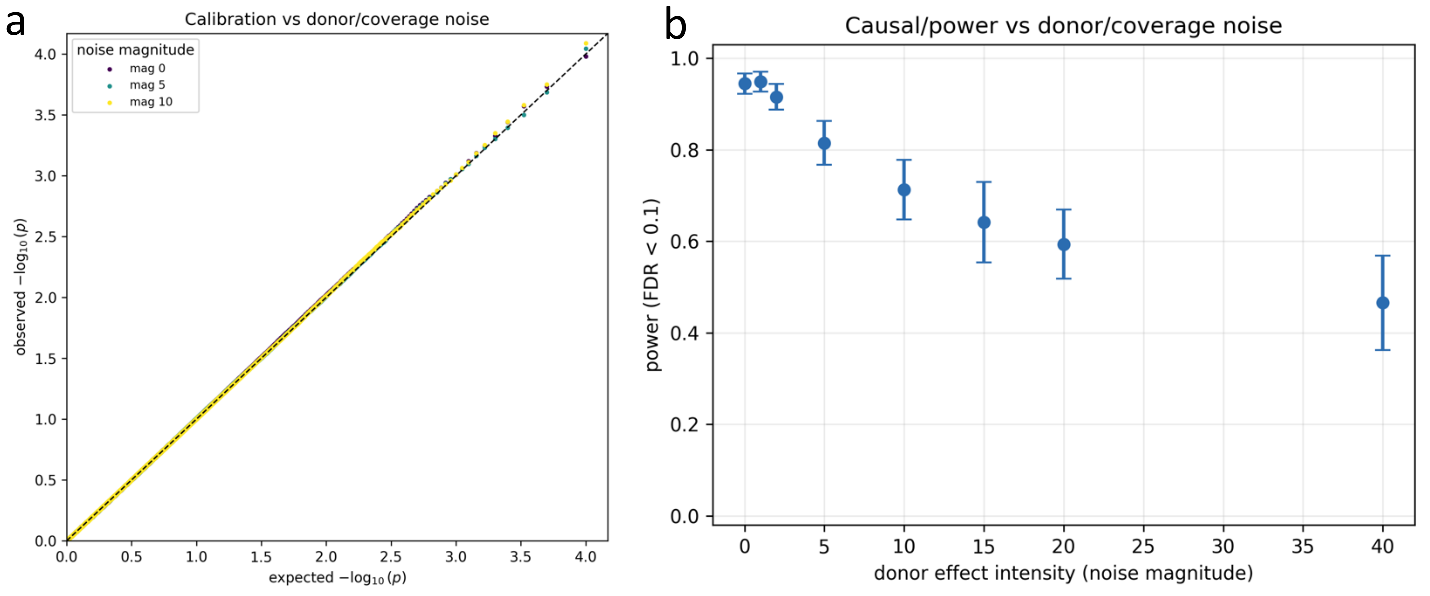

Supplementary Figure 3: Simulation with donor and coverage effect. (a) Calibration simulation with noise magnitude m of 0, 5, and 10, shown in different colors. X-axis plots the expected -log(p) and y-axis plots the observed -log(p). Each dot represents the average value across the p-value bins. (b) Causal simulation shows the power with noise magnitude m of 0, 1, 2, 5, 10, 15, 20, and 40. Each dot represents the power with an error bar of 95% confidence from standard deviation.

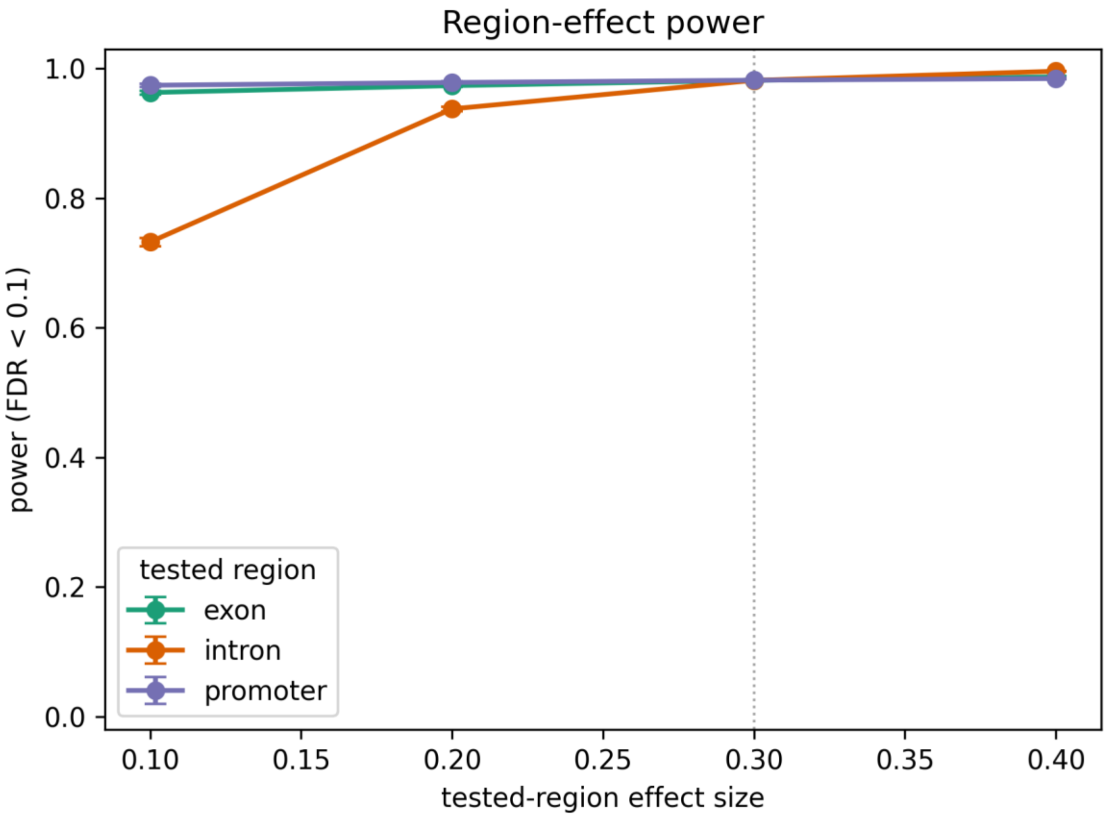

Supplementary Figure 4: Causal simulation shows power with region-effect across exon, intron, and promoter regions. Each color represents one region, where the perturbation effect changes for the tested region along the x-axis, while two untested regions have a fixed perturbation effect of 0.7.

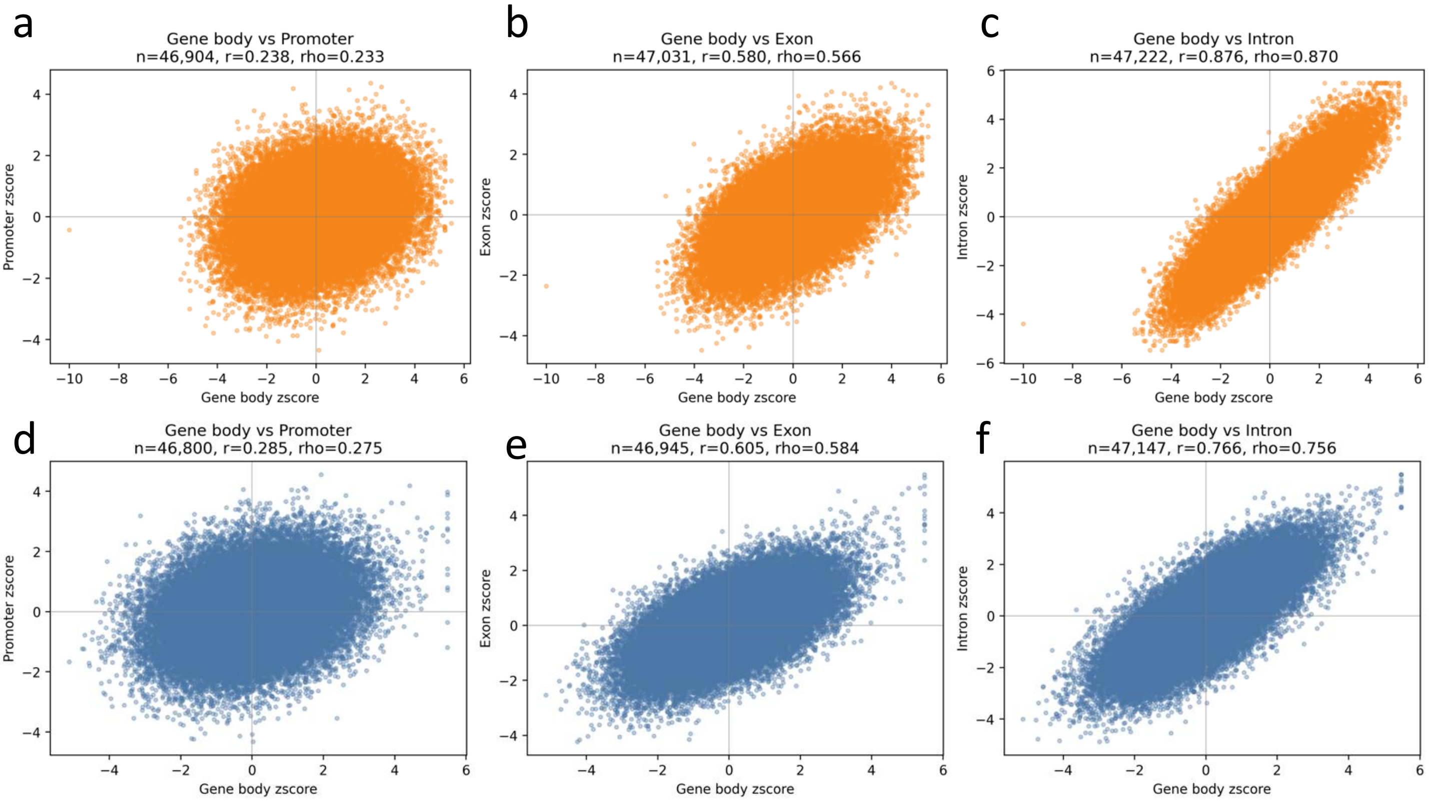

Supplementary figure 5: Scatter plot comparing the met-scDRS when methylation is aggregated by promoter (a), exons (b), introns (c) compared to methylation aggregated by gene body region for non-CpG methylation. Scatter plot comparing the met-scDRS when methylation is aggregated by promoter (d), exons (e), introns (f) compared to methylation aggregated by gene body region for CpG methylation.

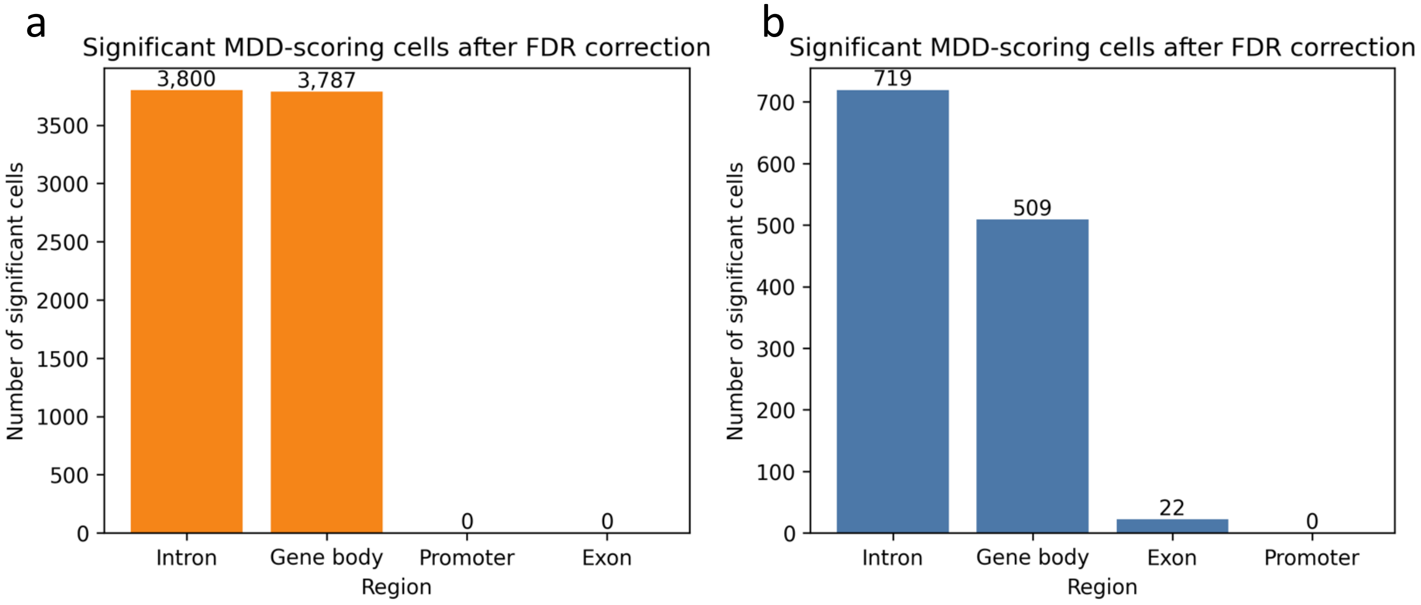

Supplementary figure 6: Bar chart showing the number of significant cells (FDR-p < 0.1) identified for MDD for non-CpG methylation (a) and CpG methylation (b) across different region aggregation methods.

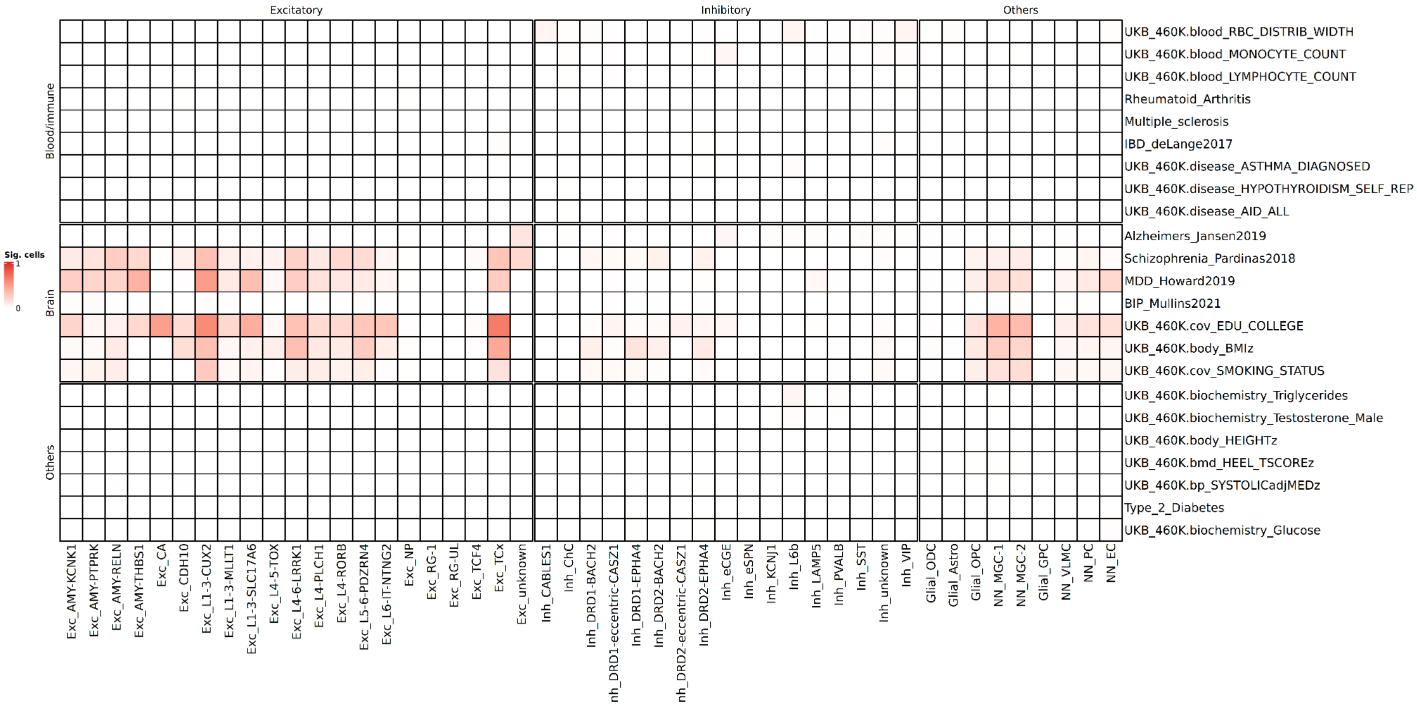

Supplementary Figure 7: Proportional heatmap showing the percent of significant (FDR-p < 0.1) cells in each cell type and trait using new QC, control scheme and covariate regression for non-CpG methylation.

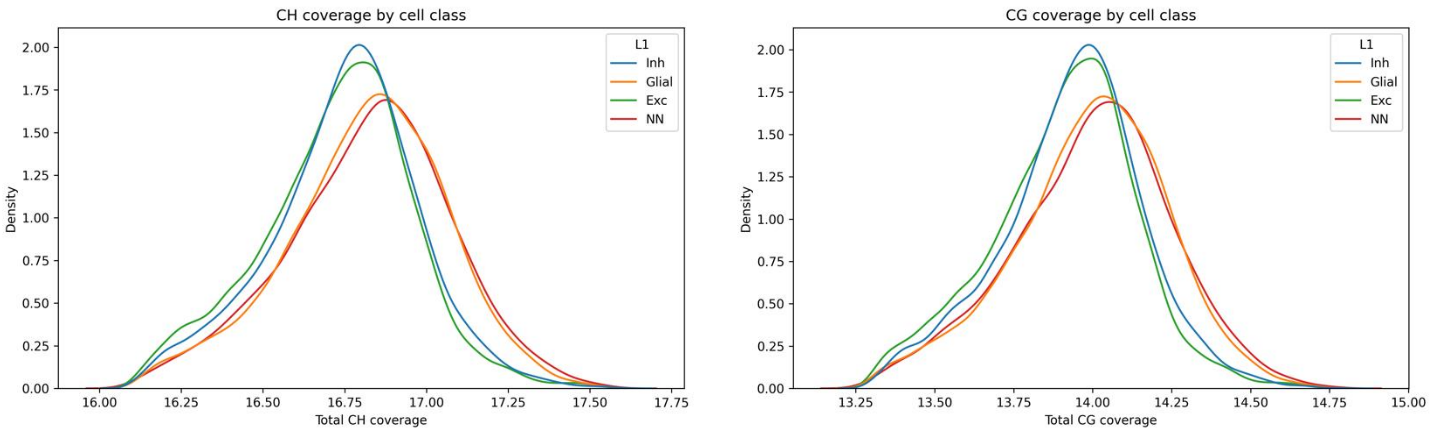

Supplementary Figure 8: Density plot showing the log-coverage colored by each cell class for non-CpG methylation (left) and CpG methylation (right).

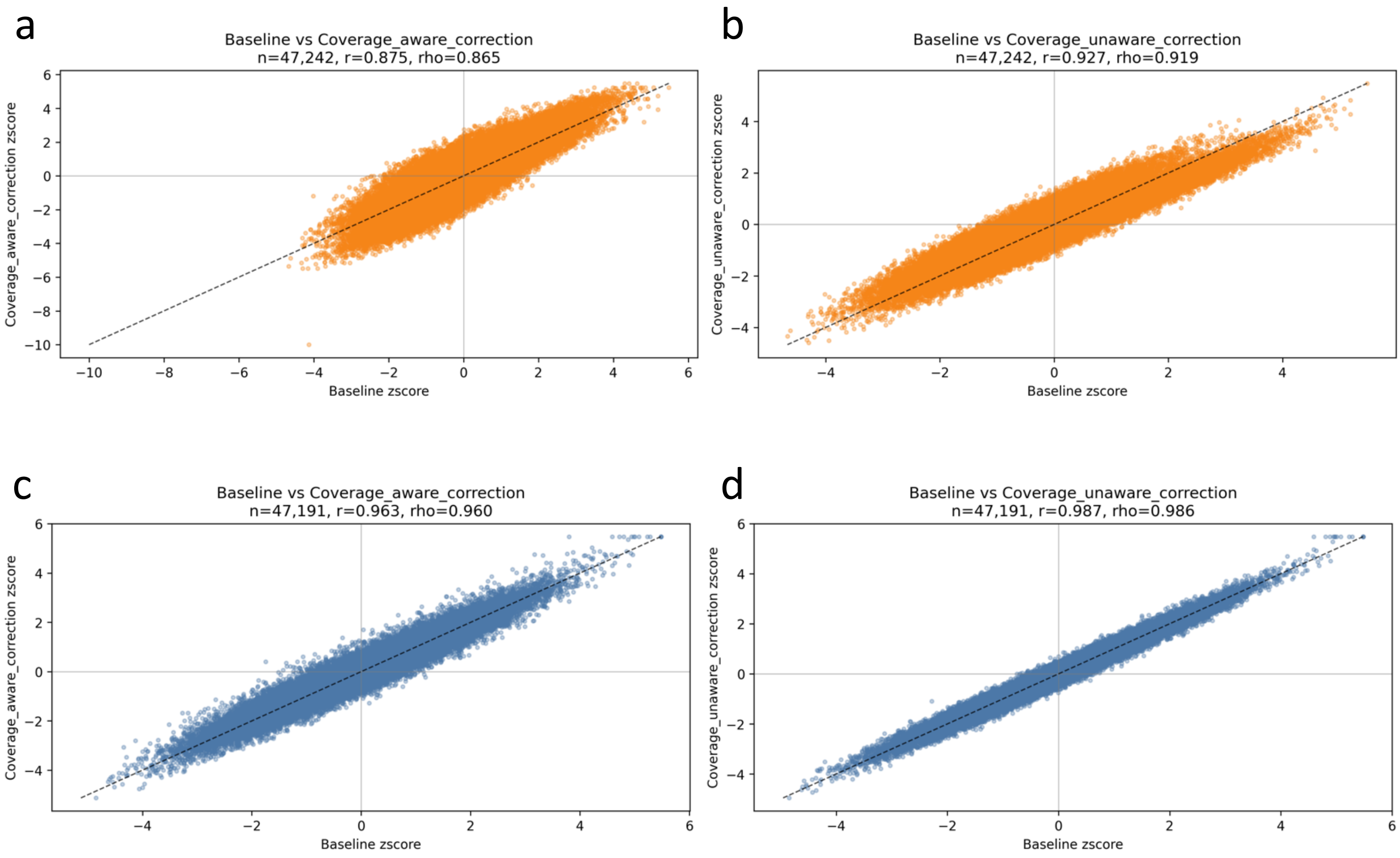

Supplementary Figure 9: Scatter plot showing met-scDRS between baseline and coverage aware correction processing (a) and coverage unaware correction (b) for non-CpG methylation. Scatter plot showing met-scDRS between baseline and coverage aware correction processing (c) and coverage unaware correction processing (d) for CpG methylation.

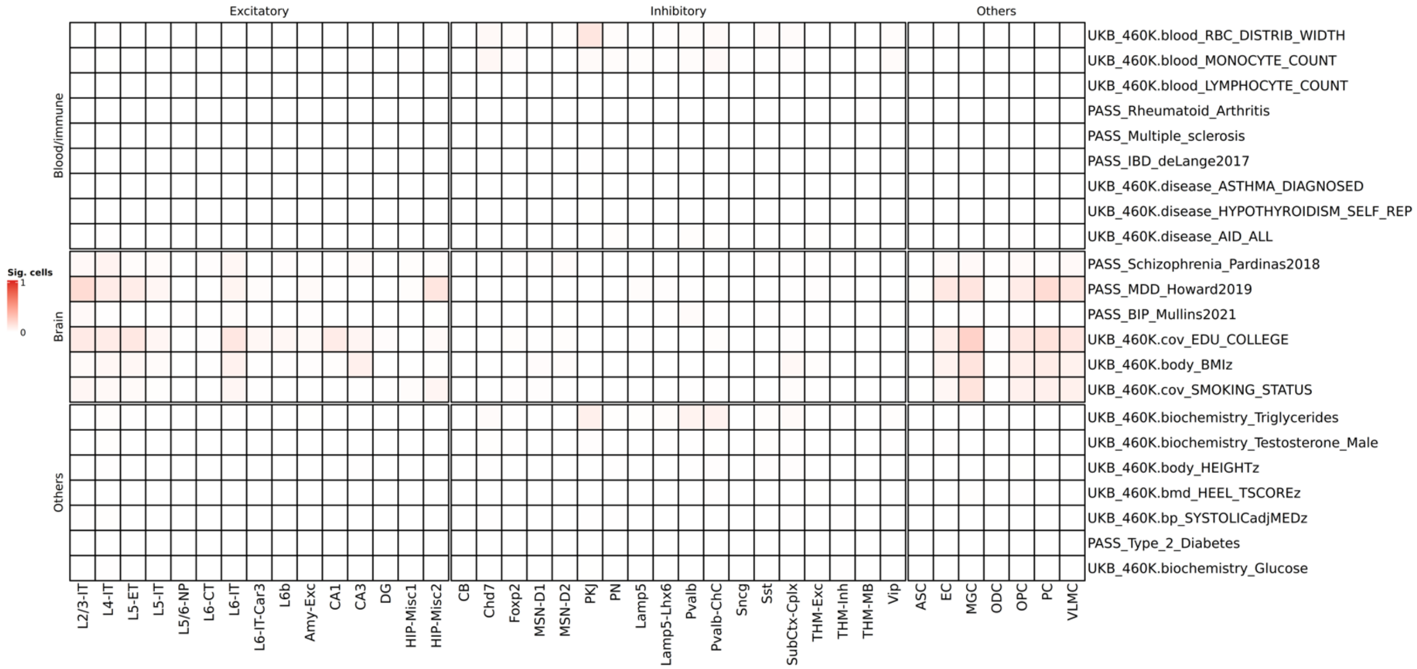

Supplementary figure 10: Heatmap showing the proportion of cells that are significant to each trait in each of the major cell type computed using non-CpG methylation modality for GSE215353 human atlas data. i.e.: cells in cell type that are significant / number of cells in each cell type. The traits in the rows are selected to represent blood / immune, brain, and other traits.

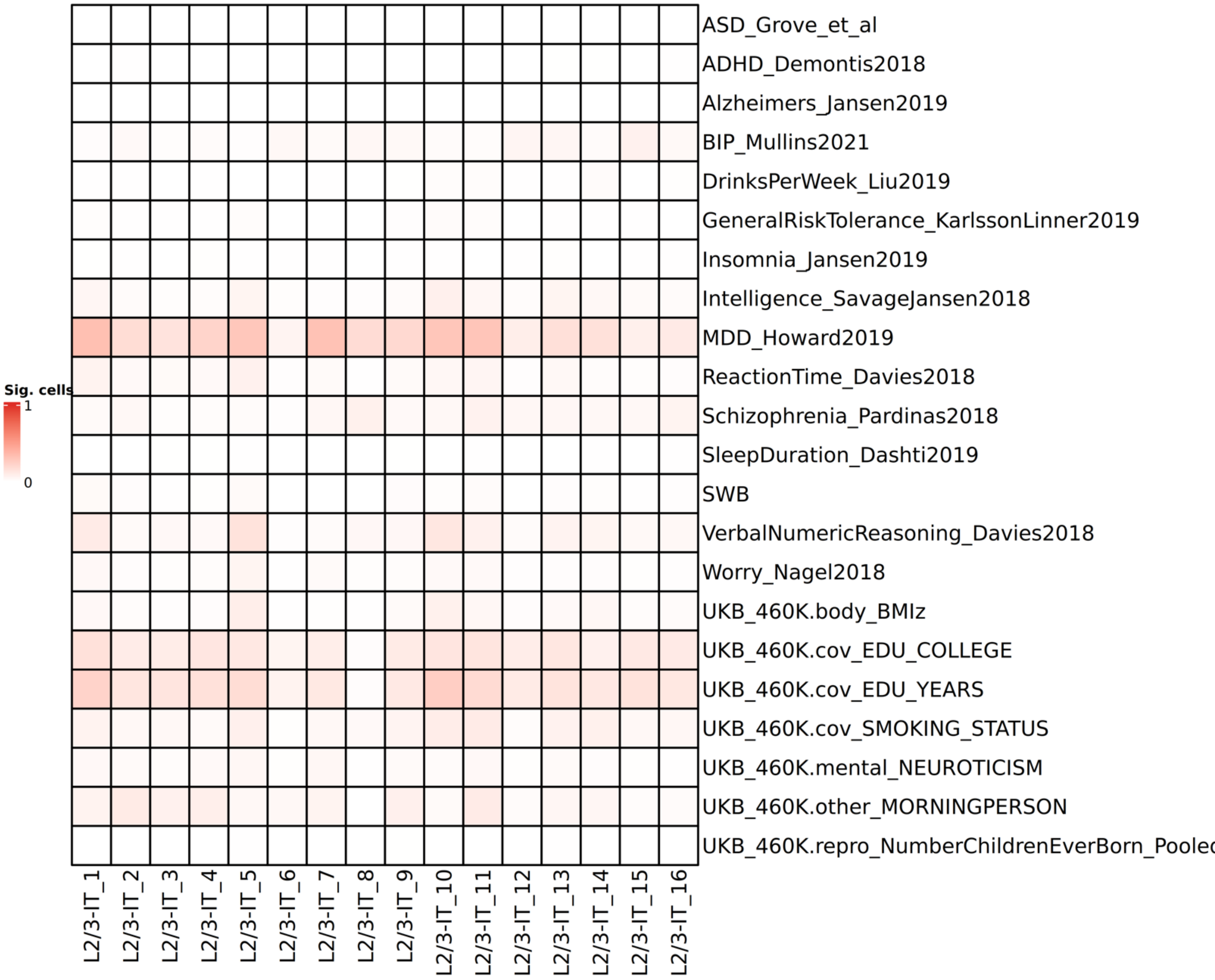

Supplementary figure 11: Heatmap showing the proportion of cells in each of the L2/3-IT neuronal subtypes that are significant in each of the traits.

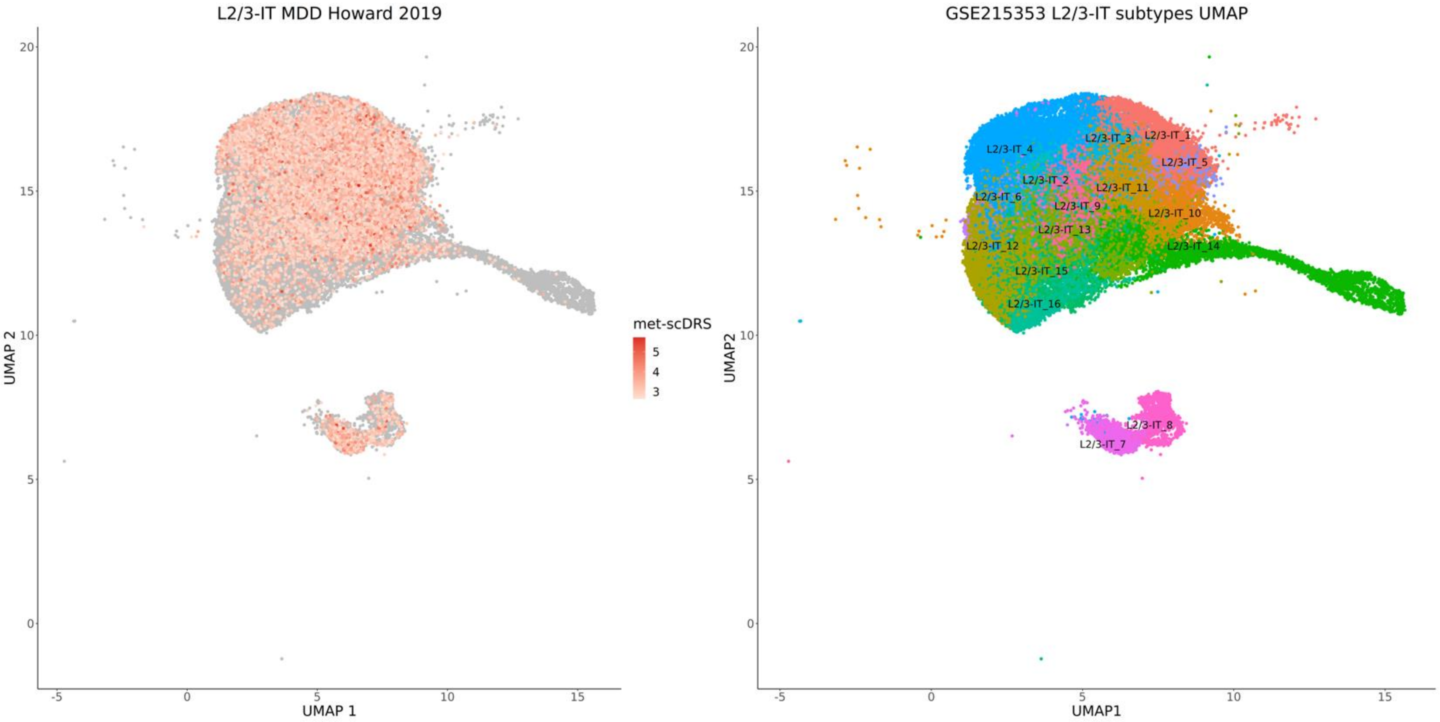

Supplementary figure 12: UMAP plot colored by met-scDRS for human atlas L2/3-IT neurons for MDD and b) UMAP plot colored by cell types, significant cells post correction are colored from white to red and in-significant cells colored in grey

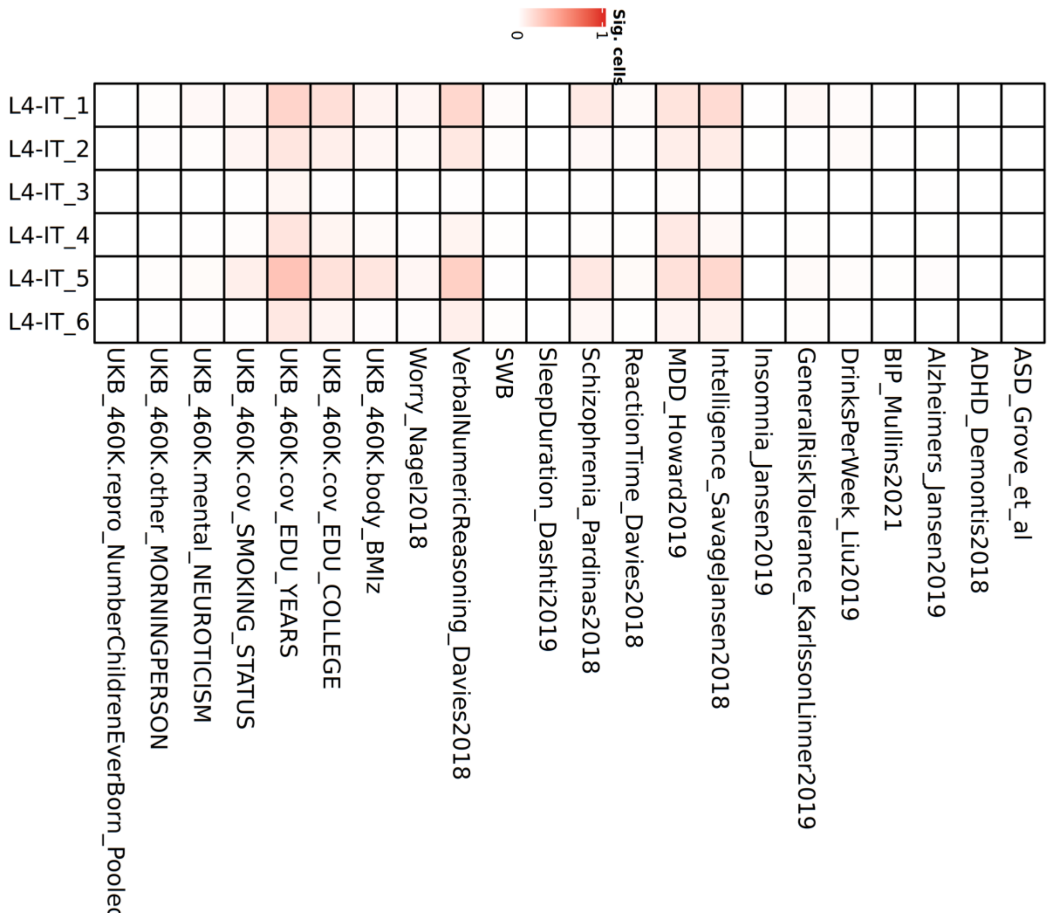

Supplementary figure 13: Heatmap showing the proportion of cells in each of the L4-IT neuronal subtypes that are significant in each of the traits.

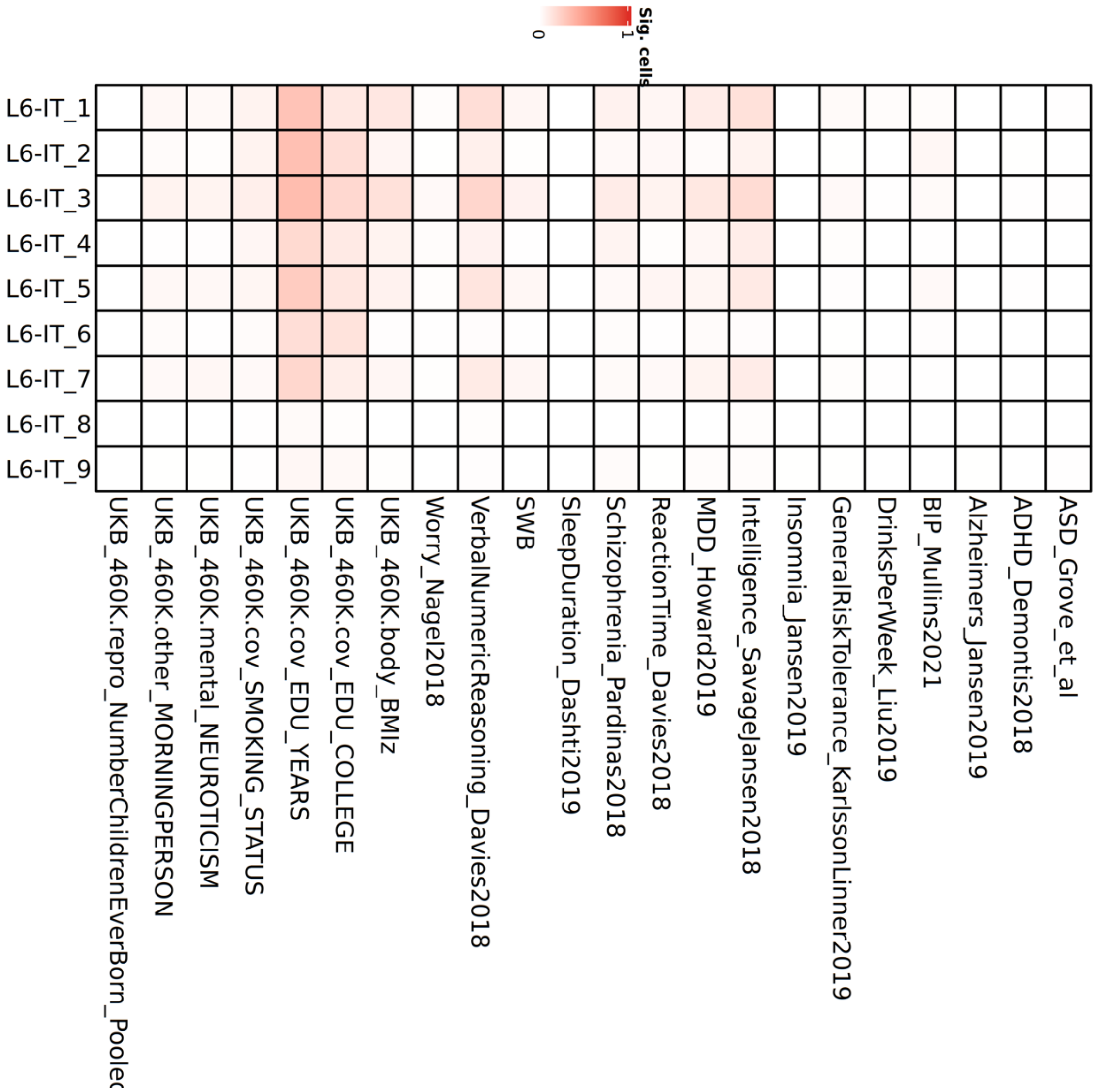

Supplementary figure 14: Heatmap showing the proportion of cells in each of the L6-IT neuronal subtypes that are significant in each of the traits.

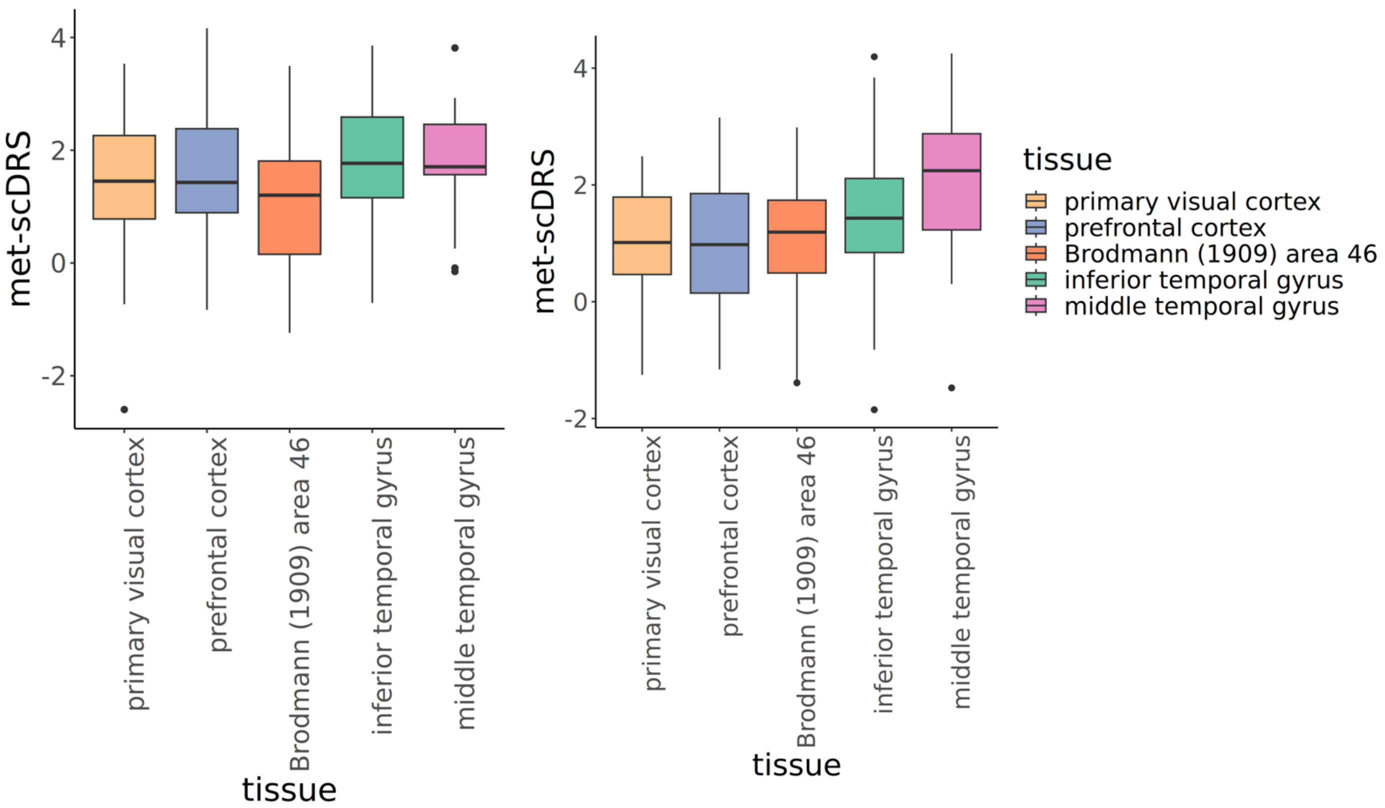

Supplementary figure 15: box plot showing the distribution of MDD met-scDRS among MGC cell type (left) and OPC cell type (right) across brain dissection regions.

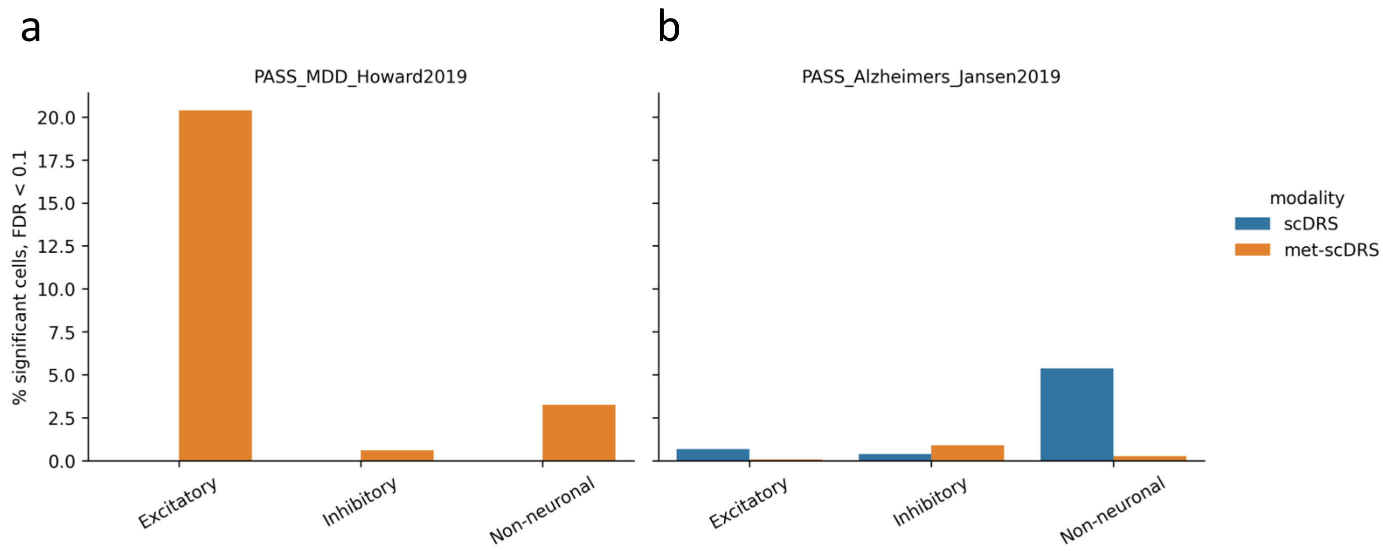

Supplementary 16: Bar chart showing the proportion of cells that are significant (FDR $p < 0.1$) for major depression disorder (a), and Alzheimer's disorder (b) in excitatory, inhibitory and non-neuronal cell class. The bar chart is colored by scDRS and met-scDRS respectively.

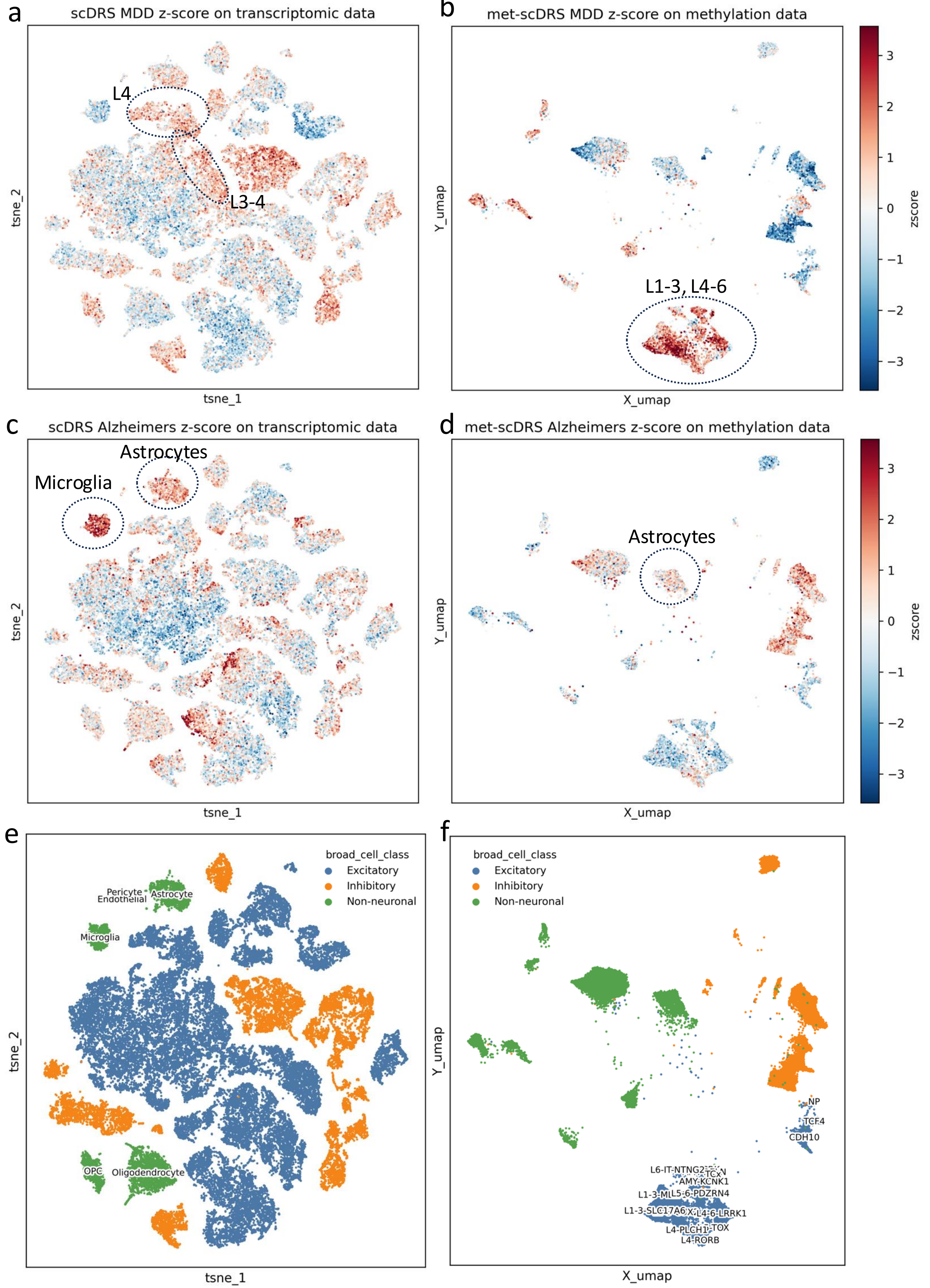

a
scDRS MDD z-score on transcriptomic data
L4
L3-4
tsne_2
tsne_1
b
met-scDRS MDD z-score on methylation data
L1-3, L4-6
Y_umap
X_umap
zscore
3
2
1
0
−1
−2
−3
c
scDRS Alzheimers z-score on transcriptomic data
Astrocytes
Microglia
tsne_2
tsne_1
d
met-scDRS Alzheimers z-score on methylation data
Astrocytes
Y_umap
X_umap
zscore
3
2
1
0
−1
−2
−3
e
broad_cell_class
Excitatory
Inhibitory
Non-neuronal
Pericyte
Endothelial
Astrocyte
Microglia
OPC
Oligodendrocyte
tsne_2
tsne_1
f
broad_cell_class
Excitatory
Inhibitory
Non-neuronal
NP
TCF4
CDH10
L6-IT-NTNG2
AMY-KCNK1
L5-6-PDZRN4
L1-3-SLC17A6
L4-6-LRRK1
L4-PLCH1
TOX
L4-RORB
Y_umap
X_umap

Supplementary Figure 17: (a) TSNE plot colored by scDRS computed using AIBS transcriptomics atlas data for MDD with L4 and L3-4 excitatory cell types labelled directly on the plot. (b) UMAP plot colored by met-scDRS in human methylome 50K subset data with L1-3, L4-6 excitatory cell types annotated. (c) TSNE plot colored by scDRS in Alzheimer's disease with astrocytes and microglial cell types annotated on plot. (d) UMAP plot colored by met-scDRS with astrocytes annotated on plot. (e) TSNE plot of AIBS transcriptomics data colored by cell class. (f) UMAP plot colored by cell class in human methylome 50K subset data.

a b

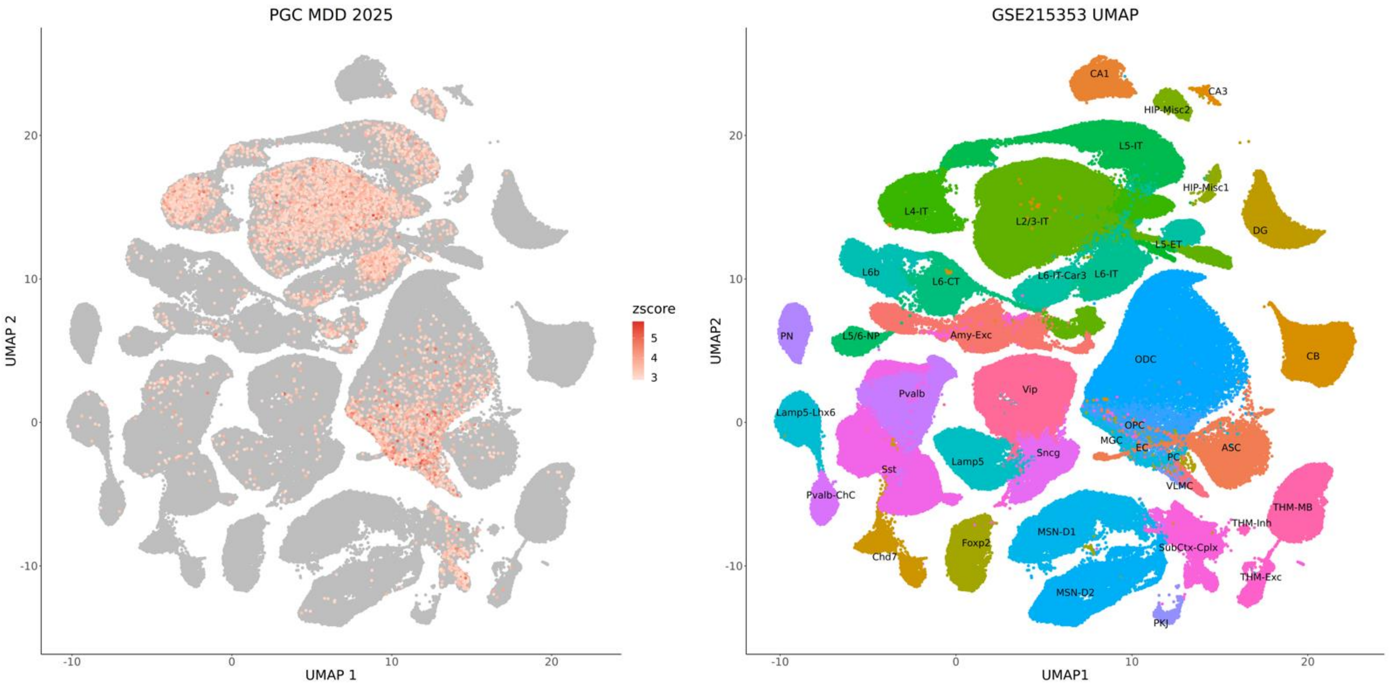

Supplementary figure 18: (a) UMAP visualization using all cells in GSE215353 for MDD trait (Adams, Mark J., et al., 2025) colored by non-CpG met-scDRS. The significant cells (FDR < 0.1) are colored with a gradient from white to red and the non-significant cells are colored as grey. (b) UMAP colored by different cell types for human atlas dataset.

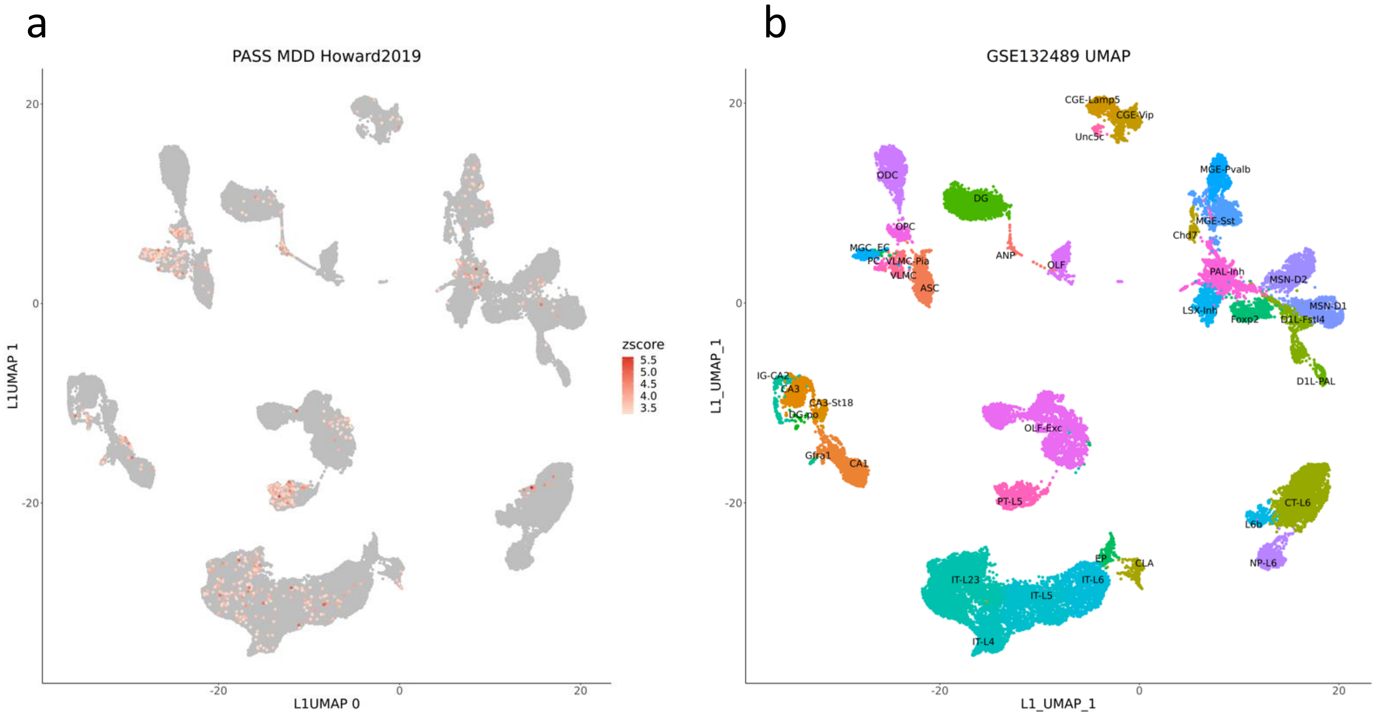

Supplementary figure 19: (a) UMAP plot colored by met-scDRS for GSE132489 mouse atlas neurons for MDD and (b) UMAP plot colored by cell types

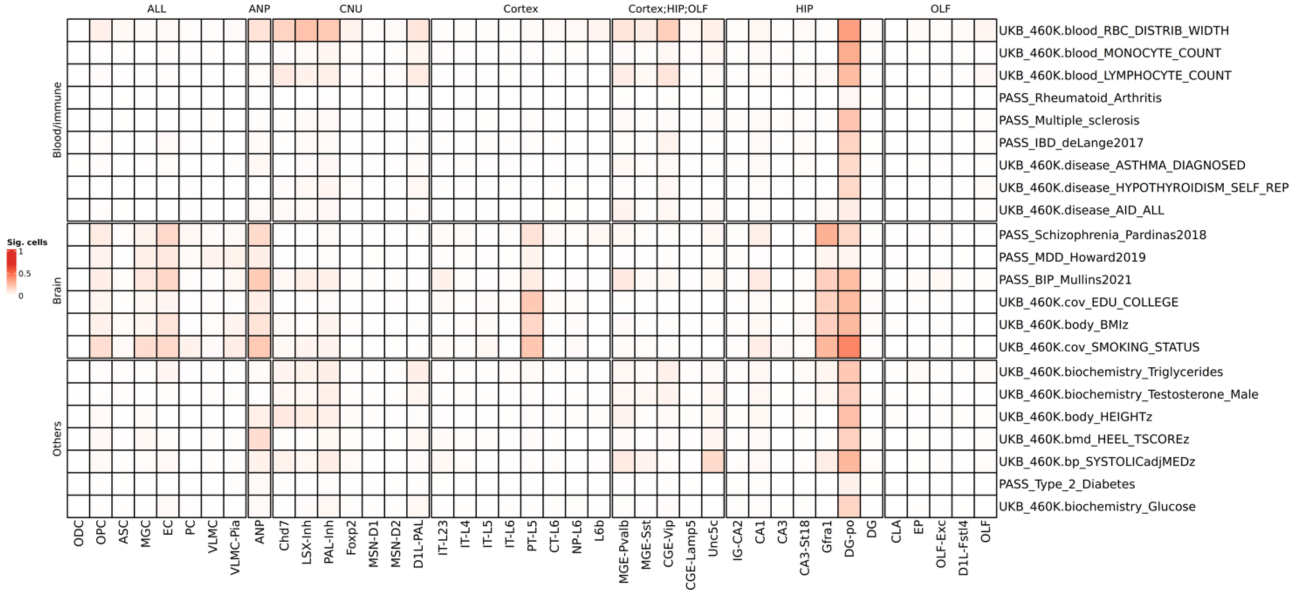

Supplementary figure 20: Heatmap showing the proportion of cells that are significant to each trait in each of the cell class computed using non-CpG methylation modality for mouse atlas gse132489 full dataset. i.e.: cells in cell type that are significant / number of cells in each cell type. The traits in the rows are selected to represent blood / immune, brain, and other traits.

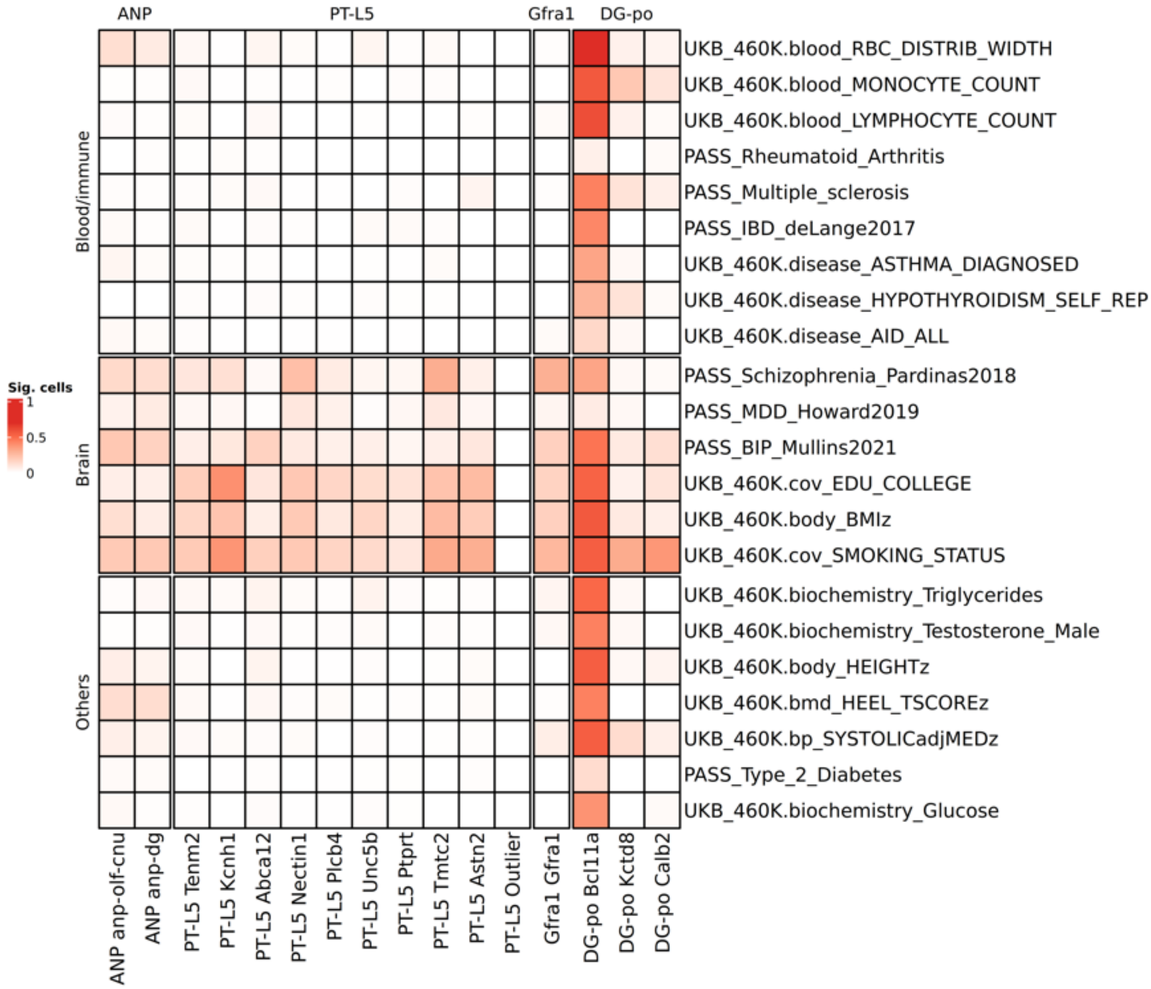

Supplementary figure 21: Proportion of significant cells among sub-cell types in GSE132489 mouse single cell methylome datasets. Only ANP, PT-L5, Gfra1 and DG-po cell types are shown for showing heterogeneity in signal associations among cell types.

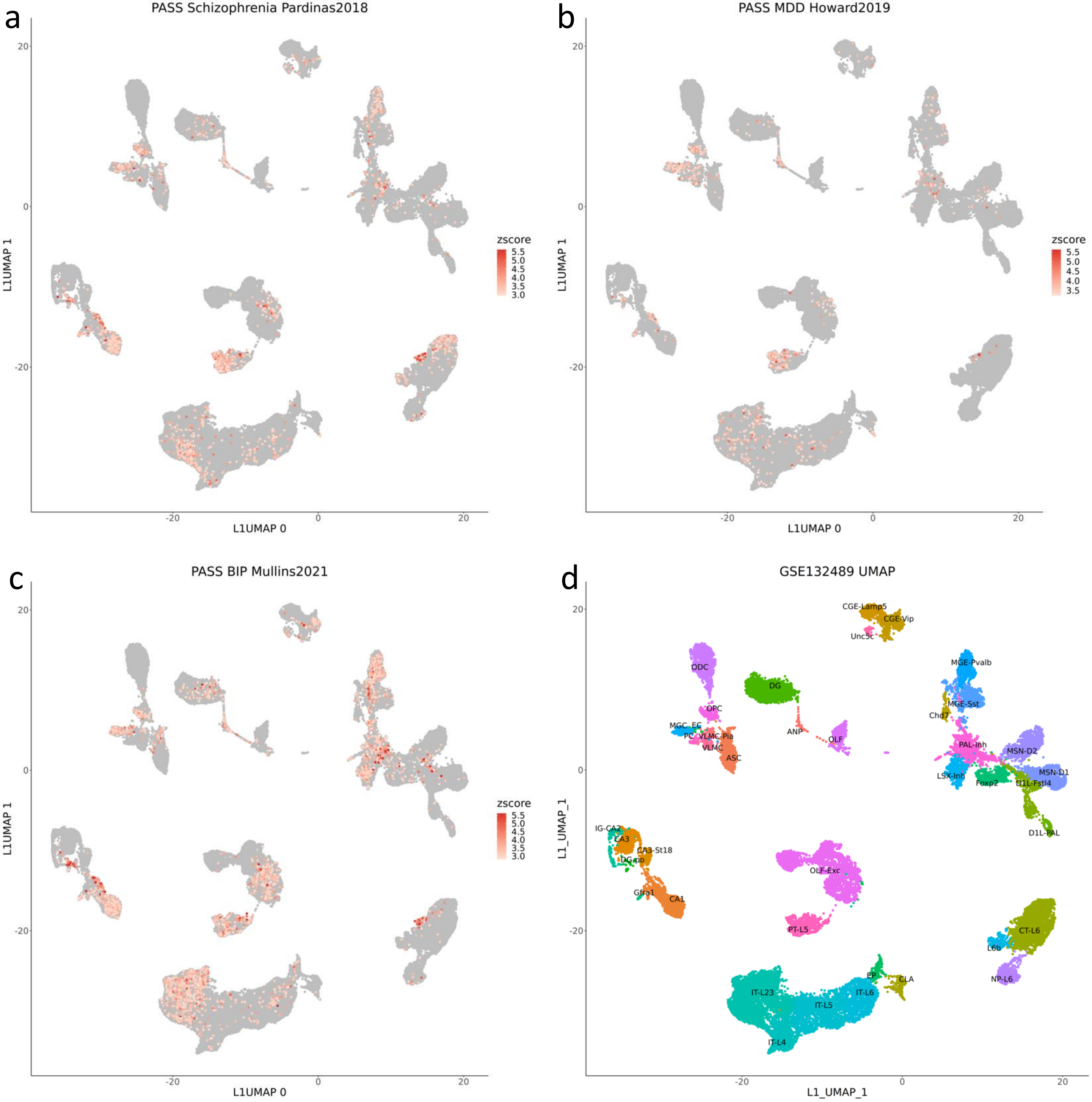

Supplementary figure 22: UMAP plot showing met-scDRS for each cell colored from white to red for significant cells in SCZ (a), MDD (b), BP (c). Cells that do not reach significance are colored in grey. (d) UMAP colored by cell types with labels are also shown for reference.

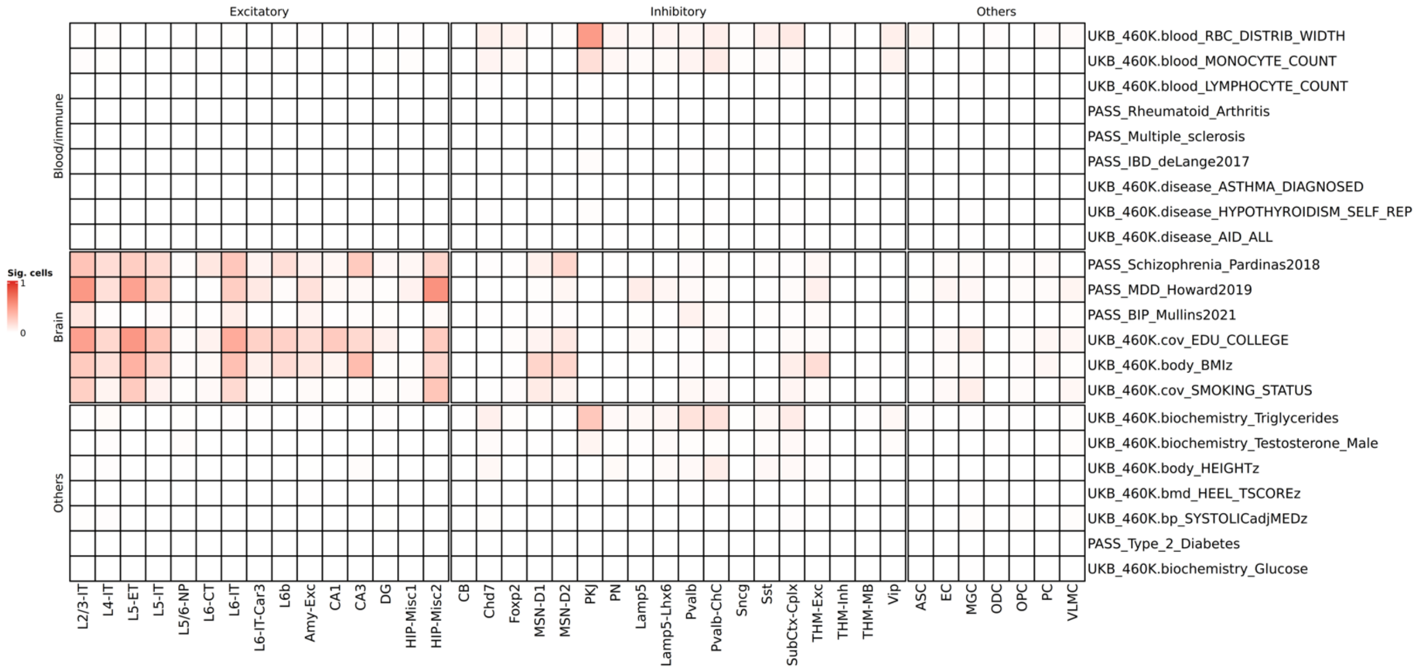

Supplementary figure 23: Heatmap showing the proportion of cells that are significant to each trait in each of the cell class computed using non-CpG methylation modality for human atlas 75K subseted dataset. i.e.: cells in cell type that are significant / number of cells in each cell type. The traits in the rows are selected to represent blood / immune, brain, and other traits. Control genes are selected by matching mean and variance only.

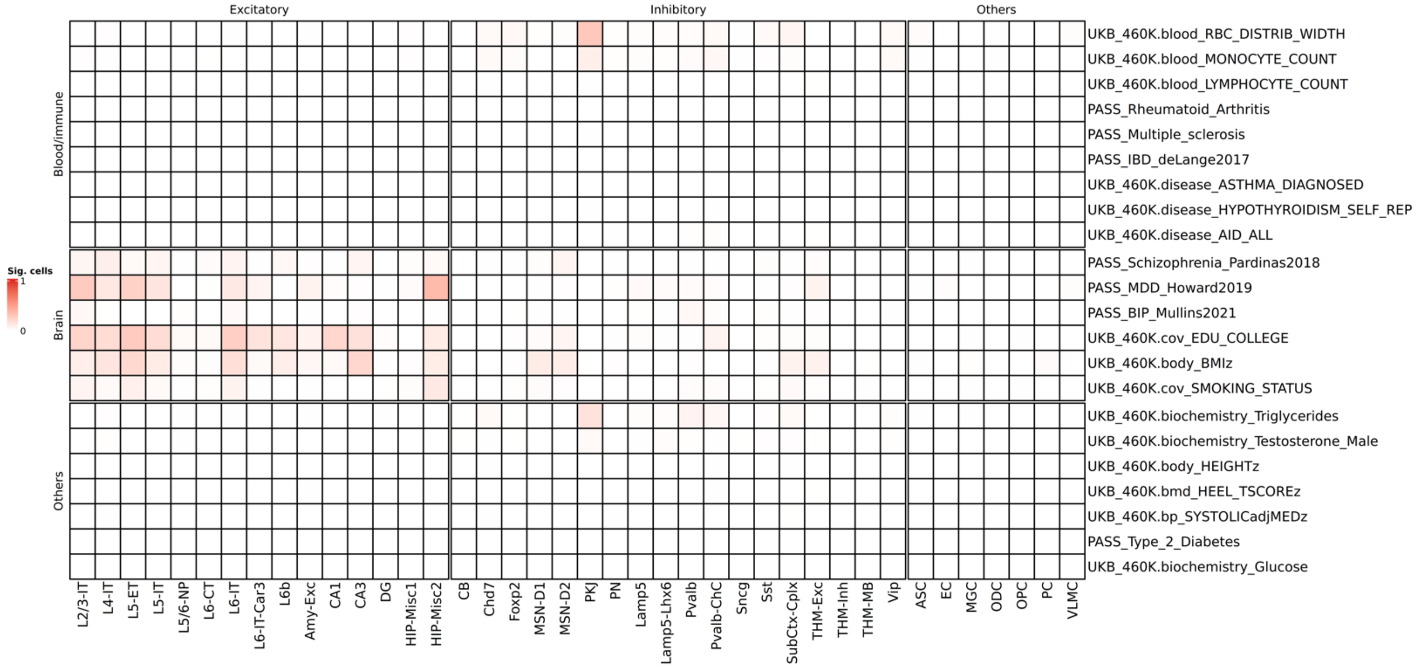

Supplementary figure 24: Heatmap showing the proportion of cells that are significant to each trait in each of the cell class computed using non-CpG methylation modality for human atlas 75K subseted dataset. i.e.: cells in cell type that are significant / number of cells in each cell type. The traits in the rows are selected to represent blood / immune, brain, and other traits. Control genes are selected by matching mean, variance and gene length

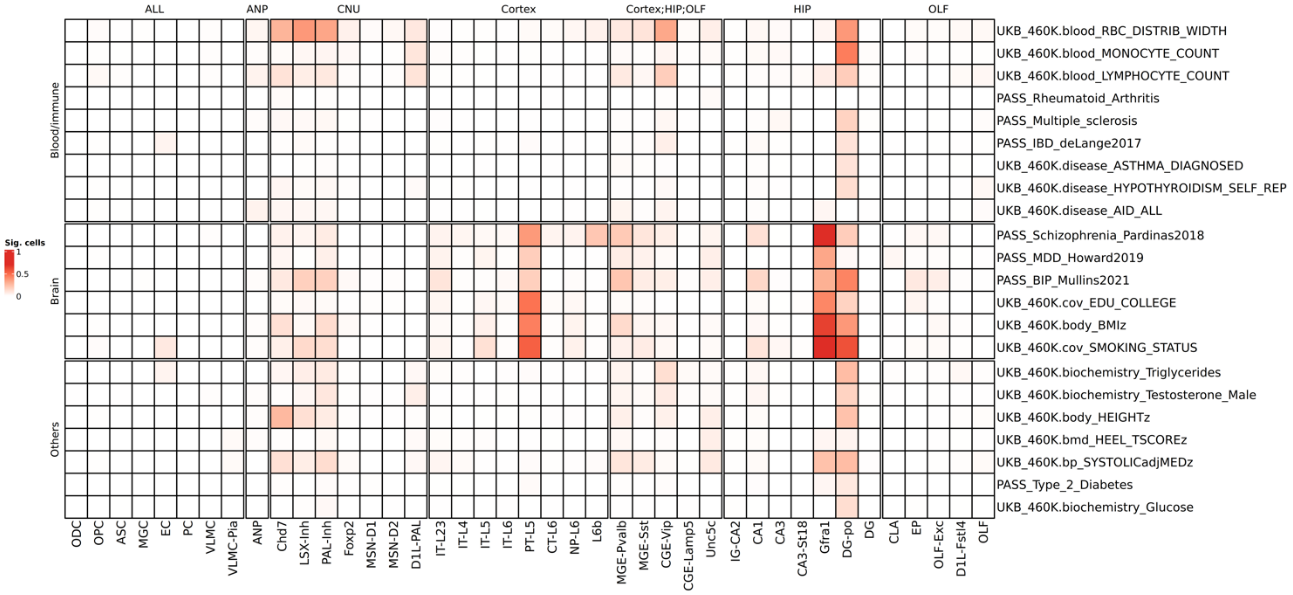

Supplementary figure 25: Heatmap showing the proportion of cells that are significant to each trait in each of the cell class computed using non-CpG methylation modality for mouse atlas 30K subseted dataset. i.e.: cells in cell type that are significant / number of cells in each cell type. The traits in the rows are selected to represent blood / immune, brain, and other traits. Control genes are selected by matching mean, variance and gene length

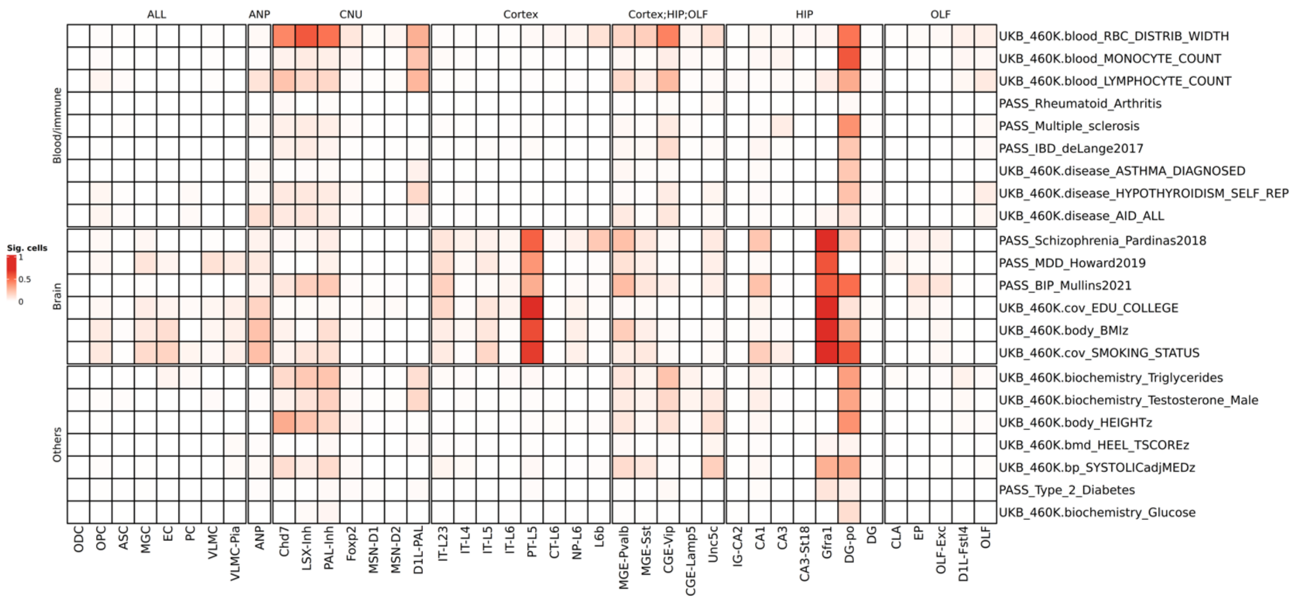

Supplementary figure 26: Heatmap showing the proportion of cells that are significant to each trait in each of the cell class computed using non-CpG methylation modality for mouse atlas 30K subseted dataset. i.e.: cells in cell type that are significant / number of cells in each cell type. The traits in the rows are selected to represent blood / immune, brain, and other traits. Control genes are selected by matching mean, variance only

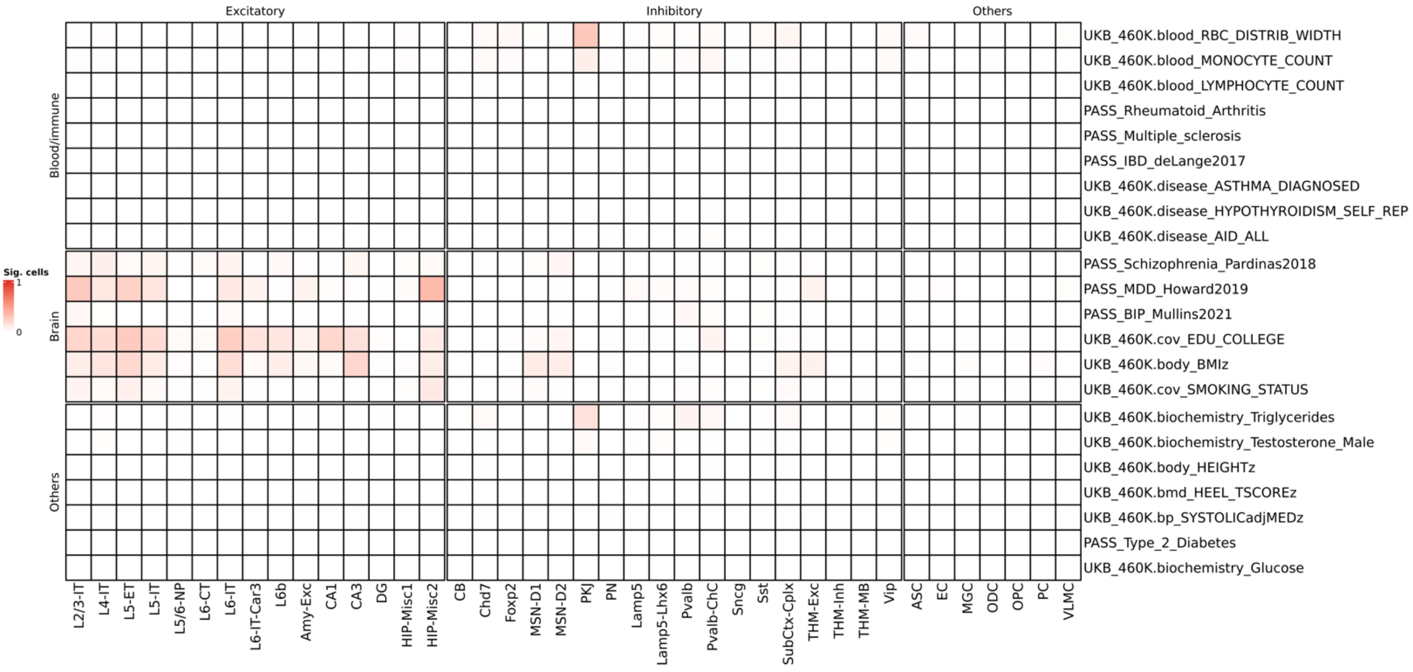

Supplementary figure 27: Proportion of significant cells in subset 75K human atlas data across different cell types with control genes mean, variance and length matched, and data is not normalized

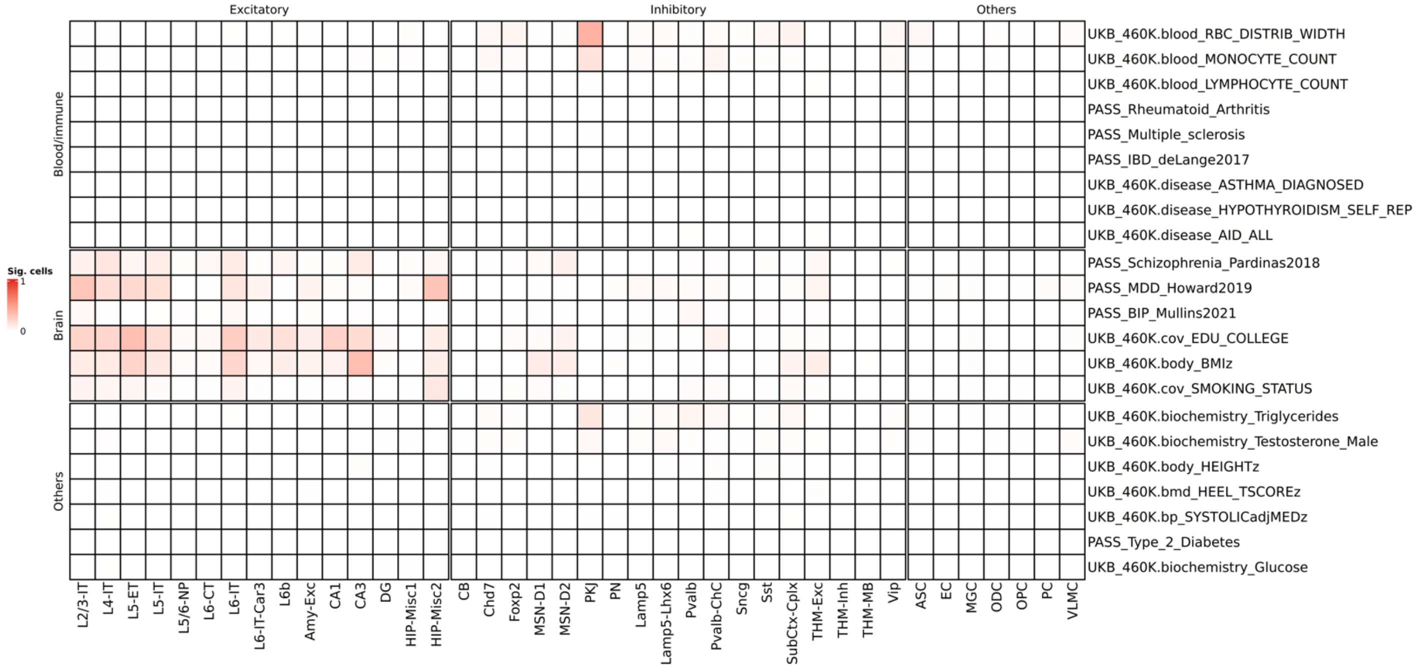

Supplementary figure 28: Proportion of significant cells in subset 75K human atlas data across different cell types with control genes mean, variance and length matched, and data is normalized by arcsine transformation

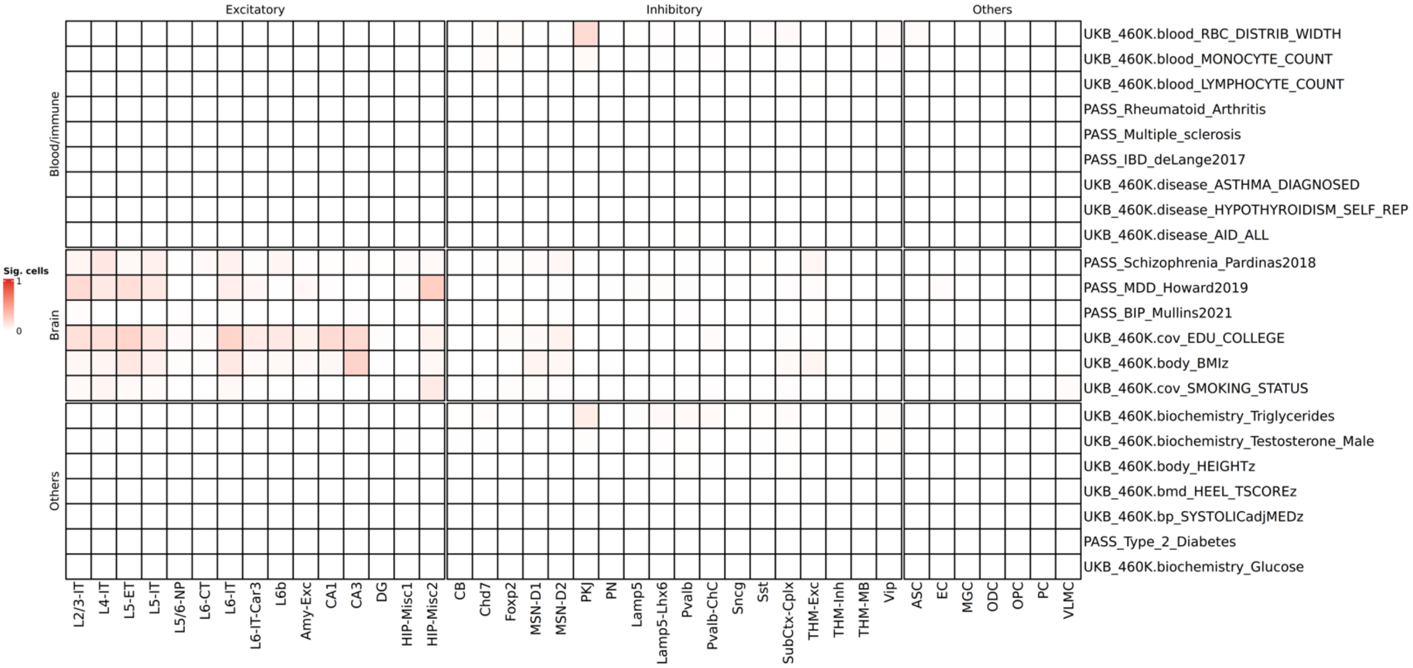

Supplementary figure 29: Proportion of significant cells in subset 75K human atlas data across different cell types with control genes mean, variance and length matched, and data is normalized by logit transformation

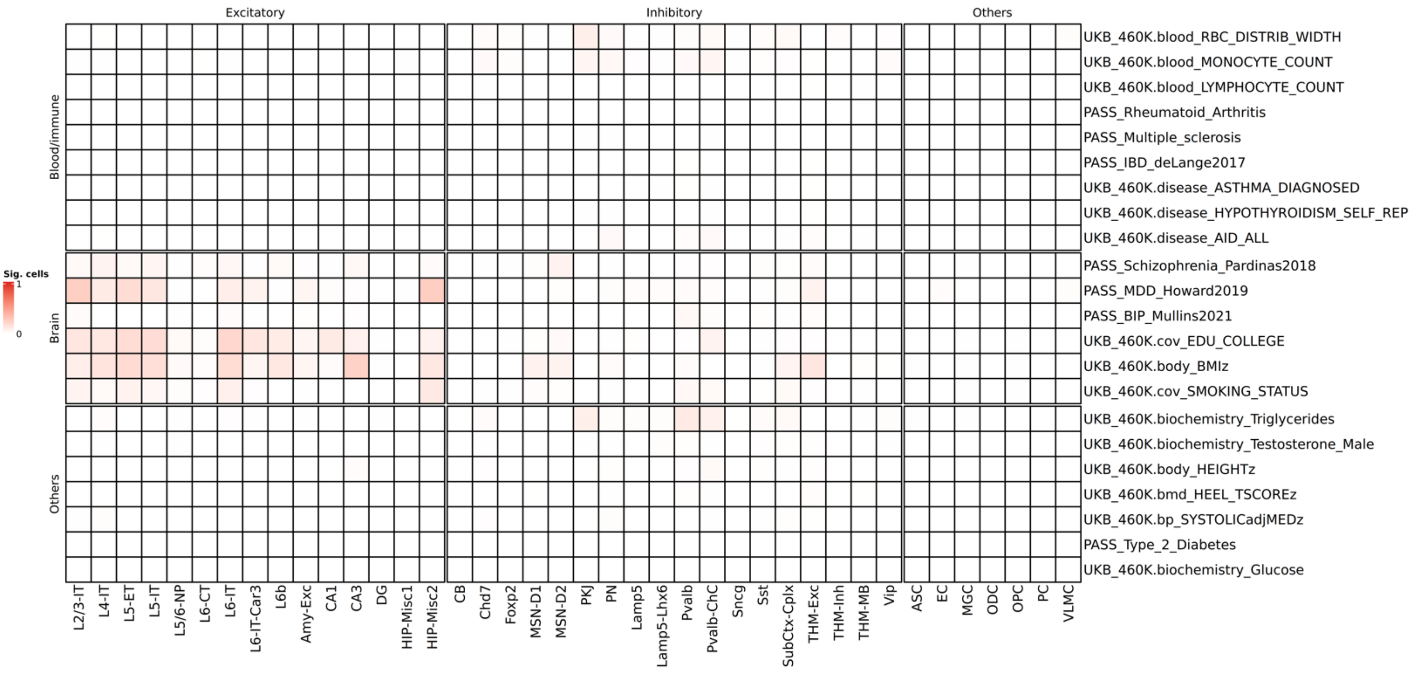

Supplementary figure 30: Proportion of significant cells in subset 75K human atlas data across different cell types with control genes mean, variance and length matched, and data is normalized by library size

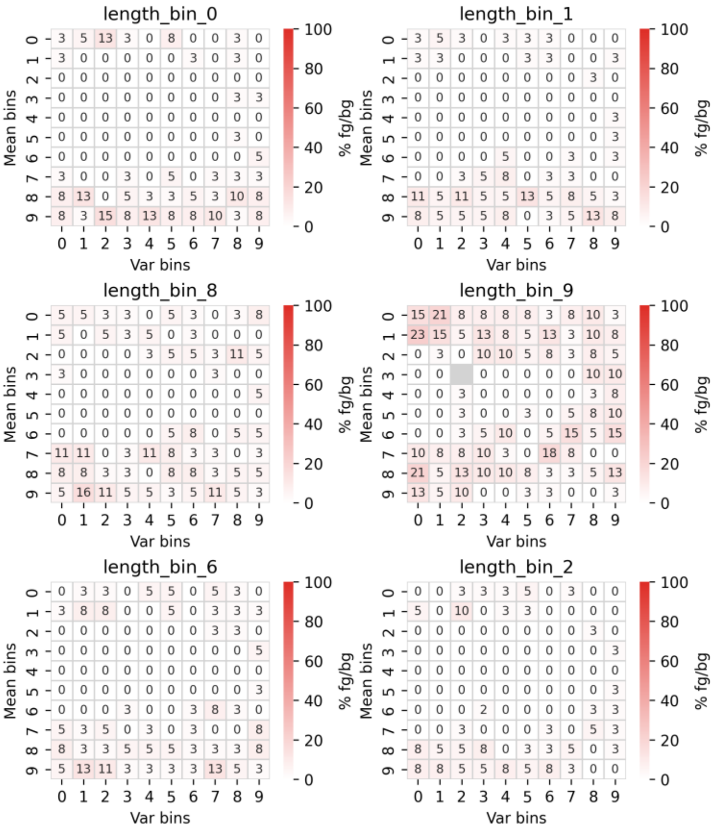

Supplementary figure 31: Heatmap showing the foreground / background ratio of genes across mean, variance and gene length bins for MDD. Gray represents bins where no genes fall into the quantile bin.

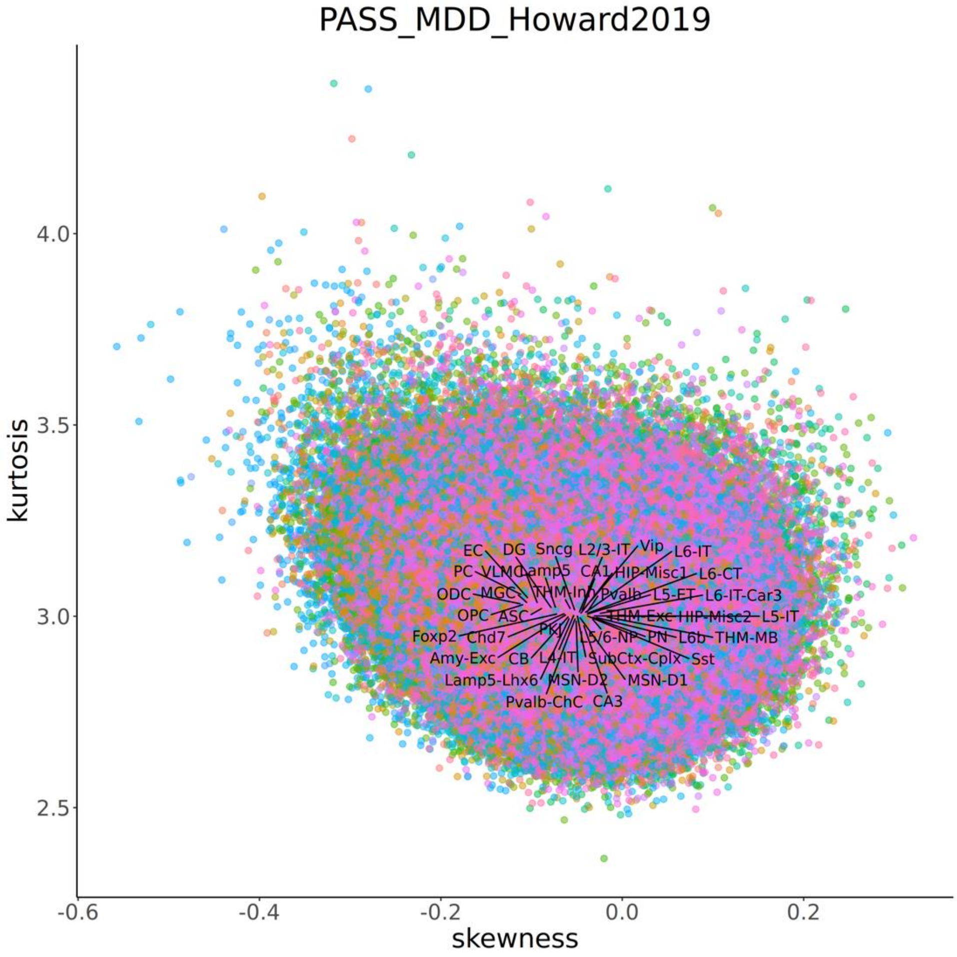

Supplementary figure 32: scatter plot showing the kurtosis and skewness to each of the null distributions for MDD colored by cell types. Cell type labels are directly specified on graph as the center location for kurtosis and skewness for each cell type.

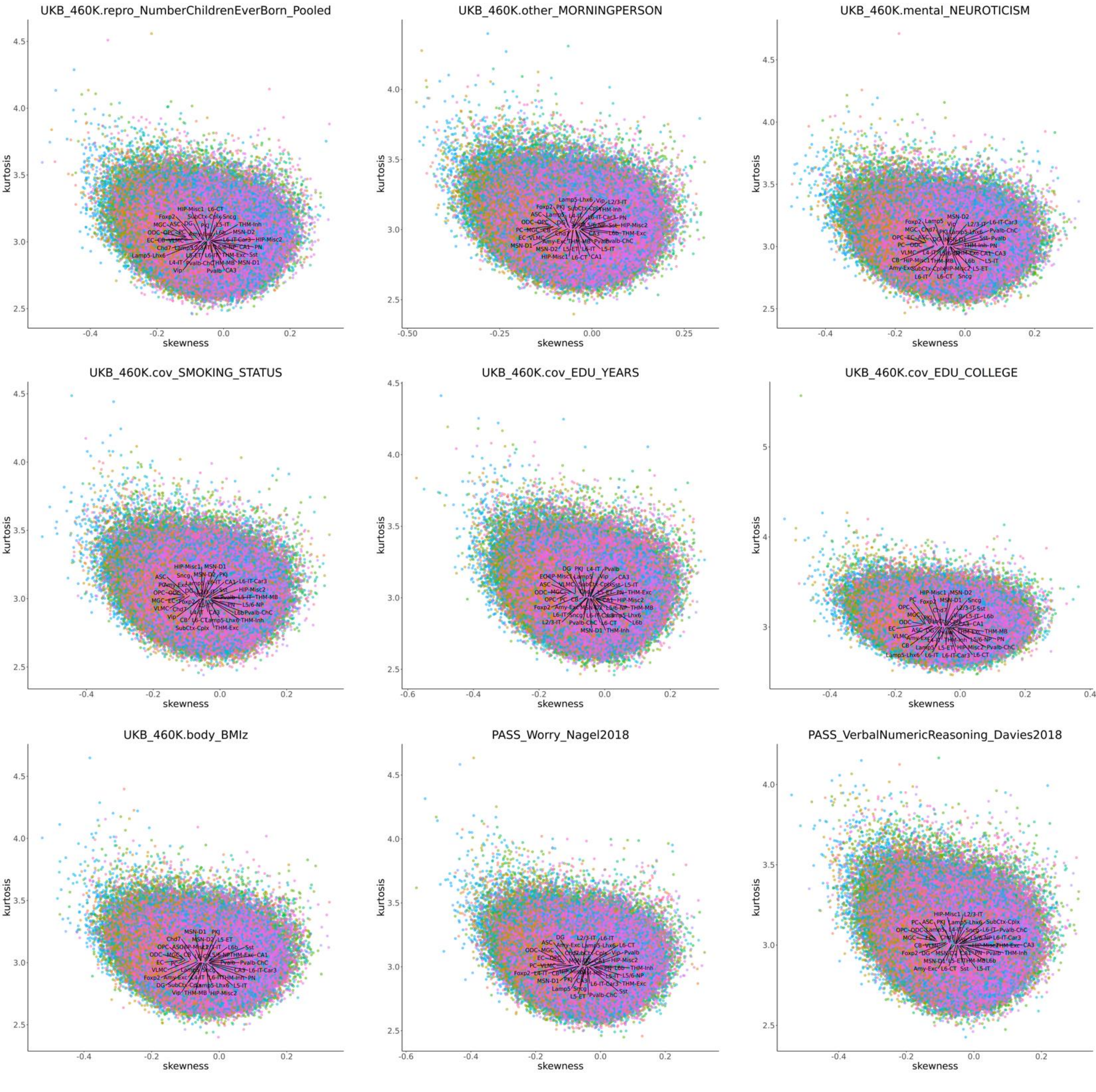
UKB_460K.repro_NumberChildrenEverBorn_Pooled
UKB_460K.other_MORNINGPERSON
UKB_460K.mental_NEUROTICISM
UKB_460K.cov_SMOKING_STATUS
UKB_460K.cov_EDU_YEARS
UKB_460K.cov_EDU_COLLEGE
UKB_460K.body_BMIz
PASS_Worry_Nagel2018
PASS_VerbalNumericReasoning_Davies2018
kurtosis
skewness

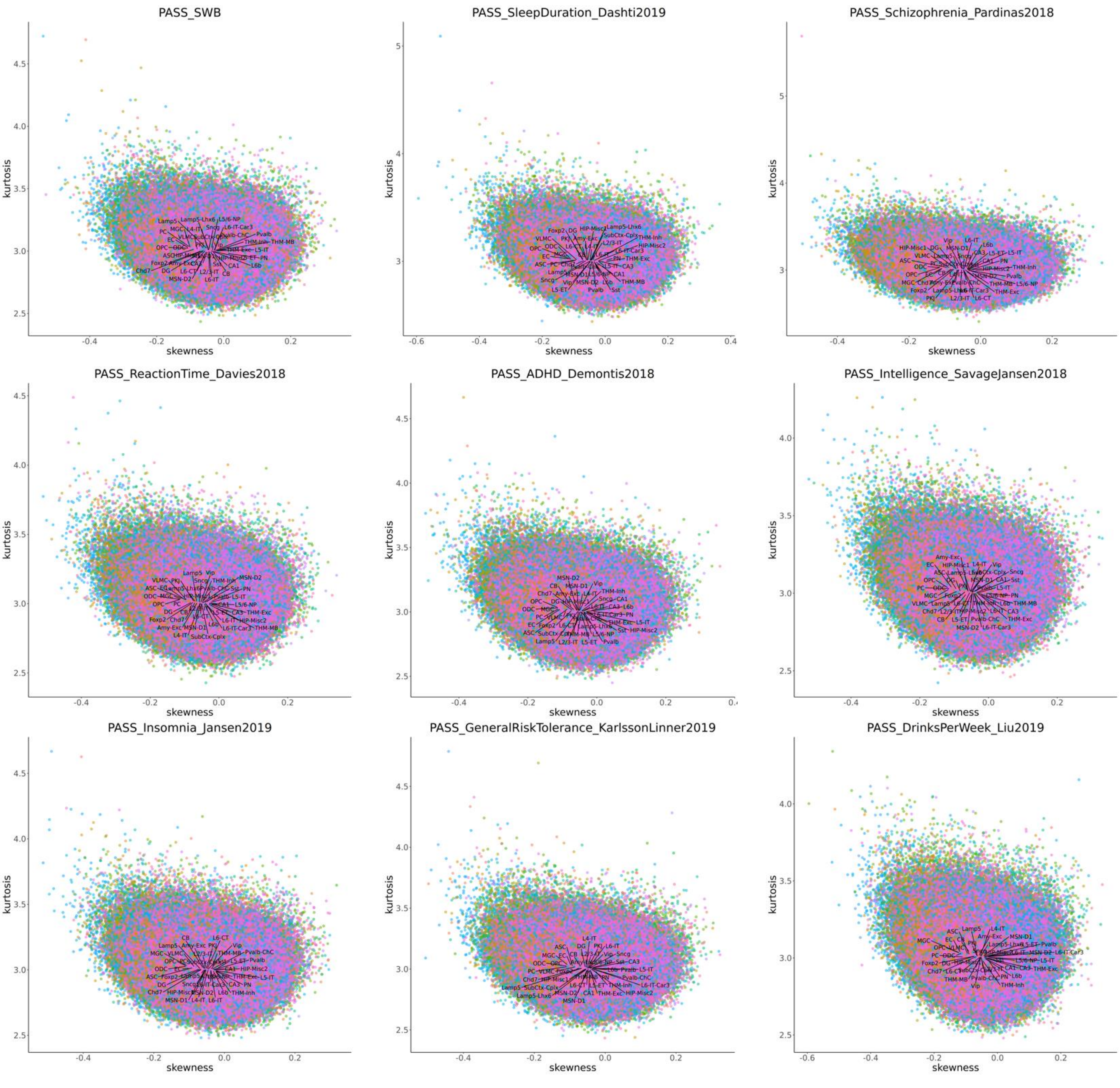
PASS_SWB
PASS_SleepDuration_Dashti2019
PASS_Schizophrenia_Pardinas2018
PASS_ReactionTime_Davies2018
PASS_ADHD_Demontis2018
PASS_Intelligence_SavageJansen2018
PASS_Insomnia_Jansen2019
PASS_GeneralRiskTolerance_KarlssonLinner2019
PASS_DrinksPerWeek_Liu2019
kurtosis
skewness

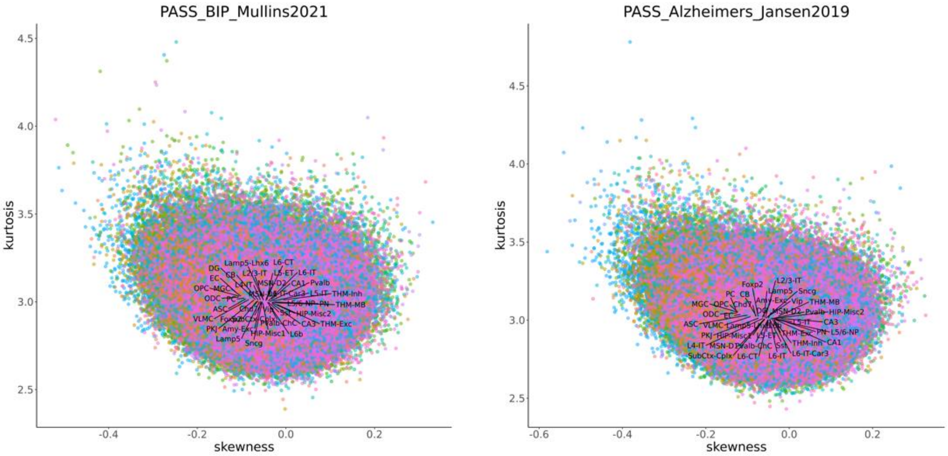

Supplementary figure 33: scatter plot showing the kurtosis and skewness to each of the null distributions for all brain traits except MDD colored by cell types. Cell type labels are directly specified on graph as the center location for kurtosis and skewness for each cell type.
